# Reframing Partial Root-Zone Irrigation: A Spatial Stress-Priming Mechanism for Crop Adaptation to Abiotic Stresses

**DOI:** 10.3390/plants15111714

**Published:** 2026-06-01

**Authors:** Junjie Liu, Fasih Ullah Haider, Yujia Liu, Peng Zhang, Tianhao Liu, Xiangnan Li, Sien Li

**Affiliations:** 1 Center for Agricultural Water Research in China, China Agricultural University, Beijing 100083, China; 2023309080407@cau.edu.cn; 2 Key Laboratory of Black Soil Conservation and Utilization, Northeast Institute of Geography and Agroecology, Chinese Academy of Sciences, Changchun 130102, China; haider281@iga.ac.cn (F.U.H.); liuyujia@iga.ac.cn (Y.L.); zhangpeng06@iga.ac.cn (P.Z.); liutianhao@iga.ac.cn (T.L.); 3 National Field Scientific Observation and Research Station on Efficient Water Use of Oasis Agriculture in Wuwei of Gansu Province, Wuwei 733009, China

**Keywords:** root-zone heterogeneity, stress acclimation, root-to-shoot signaling, rhizosphere microbiome, redox homeostasis

## Abstract

Abiotic stresses limit crop productivity by disrupting water relations, carbon assimilation, nutrient acquisition, membrane stability, and redox homeostasis. Partial root-zone irrigation (PRI), commonly implemented as partial root-zone drying (PRD), is often viewed as a deficit-irrigation strategy to improve water-use efficiency; however, this view underestimates the biological consequences of spatial root-zone heterogeneity. This review evaluates PRI as a spatially structured, priming-like framework for crop adaptation to abiotic stress. Available evidence indicates that localized drying and wet-side water uptake can coordinate root sensing, hydraulic–chemical signaling, abscisic acid delivery, hormone crosstalk, xylem-mediated regulation, and stomatal control. Beyond gas exchange, PRI is associated with photosynthetic maintenance, osmotic adjustment, antioxidant and redox regulation, root architectural plasticity, nutrient acquisition, and metabolic reprogramming. Evidence is strongest for drought, whereas responses to low temperature, salinity, heat-associated evaporative demand, and combined stresses remain more context-dependent. Emerging work also links PRI to rhizosphere restructuring and microbiome shifts, but the causal mechanisms and field reproducibility remain unresolved. We argue that future progress requires matched PRI–deficit-irrigation comparisons, standardized switching thresholds, shared physiological and molecular readouts across crops, high-resolution root biology, and commercially realistic field validation. This framing distinguishes conserved physiological outcomes from mechanisms that may differ among crops, genotypes, and irrigation designs.

## 1. Introduction

Abiotic stresses remain among the most severe constraints on agricultural productivity because they disrupt the physiological coordination that underpins plant growth, resource capture, and yield formation [1,2]. Drought, salinity, and low temperature impair plant water relations, stomatal regulation, carbon assimilation, nutrient transport, membrane stability, and redox homeostasis, often well before obvious injury becomes visible [3]. This early sensitivity is critical from both physiological and agronomic perspectives, because many of the first responses to stress are regulatory rather than purely destructive. Foundational work on plants in drying soil showed that shoot growth, leaf expansion, and stomatal behavior can be altered even when shoot water status is only weakly perturbed, implying that roots actively perceive soil water decline and transmit this information to the shoot through long-distance signaling pathways [1,2]. This shift from a damage-centered view of drought toward a signaling-based interpretation of stress remains one of the most important conceptual advances in plant water relations. This conceptual framework has important implications for irrigation science. Conventional full irrigation can temporarily buffer stress effects, but it is increasingly difficult to sustain under intensifying pressure on freshwater resources. Deficit irrigation (DI), although widely adopted to improve water-use efficiency (WUE), typically exposes the entire root system to a relatively uniform reduction in soil water availability, which may constrain nutrient acquisition, depress photosynthesis, and reduce biomass or yield when stress persists or is developmentally mis-timed [4,5]. Previous reviews have highlighted the physiological and agronomic responses associated with deficit irrigation strategies, including regulated deficit irrigation and partial root-zone drying, in perennial fruit crops [6]. Partial root-zone irrigation (PRI), most commonly implemented as partial root-zone drying (PRD), was developed as a biologically informed alternative to this uniform-deficit model [5]. In PRI, part of the root system remains in wet soil to sustain water uptake, whereas another part is exposed to drying soil to generate root-derived signals that regulate shoot physiology [1,5]. Thus, the defining feature of PRI is not simply reduced irrigation volume, but the deliberate creation of spatial heterogeneity in root-zone water status. This distinction is fundamental because it enables drought signaling to be activated without necessarily imposing the same degree of whole-plant dehydration associated with conventional water deficit.

Early experimental studies established the physiological basis of this strategy. In grapevine (*Vitis vinifera* L.), PRD improved water-use efficiency by up to 50% without significant crop reduction and was associated with marked hormonal changes in roots and altered stomatal behavior [7]. In potato (*Solanum tuberosum* L.), PRD maintained tuber biomass comparable to full irrigation while saving 30% irrigation water and increasing crop WUE by 59% [8]. In greenhouse-grown tomato (*Solanum lycopersicum* L.), PRD was shown to be agronomically feasible under reduced water supply [9], and similar conclusions were reached for processing tomato under field conditions [10]. Importantly, the concept has not remained confined to perennial horticultural systems. Evidence from maize (*Zea mays* L.) indicates that alternate PRI can improve growth together with water and nitrogen use efficiencies under interacting soil water and fertilization regimes [11], while work in cotton (*Gossypium hirsutum* L.) has shown that PRI can modify physiology and yield formation under water-limited conditions [12]. Collectively, these studies demonstrate that PRI is not crop-restricted in principle, even though its agronomic magnitude and mechanistic expression remain species-, genotype-, and environment-dependent. However, PRI should not be reduced to a simple ABA-centered explanation. Although abscisic acid (ABA) is clearly central to root-to-shoot communication during partial soil drying, later work has shown that the PRI response depends on the interaction among xylem ABA concentration, sap flow from the drying roots, root water potential, soil physical properties, and the sensitivity of the shoot to incoming signals [13]. In sunflower (*Helianthus annuus* L.), root water potential was shown to integrate the effects of contrasting substrates on ABA signaling more consistently than soil variables alone, emphasizing that signaling strength depends not only on how dry the soil becomes, but also on whether drying roots remain hydraulically functional enough to export signals to the shoot [13]. In tomato, PRD increased xylem ABA concentration 2.5-fold and reduced whole-plant transpiration, but also decreased leaf cytokinin status by 46%, indicating that PRI responses involve broader hormonal rebalancing rather than a single-signal mechanism [14]. Likewise, in lemon (*Citrus limon* (L.) Burm. f.), PRD increased crop WUE by 83% even though enhanced performance could not be explained by increased leaf xylem ABA concentration alone [15]. These observations are consistent with broader work on root-to-shoot signaling, which shows that chemical and hydraulic processes interact rather than operate independently [16,17]. PRI is therefore better understood as a structured signaling environment, in which root-zone heterogeneity alters the quantitative and qualitative nature of the information transmitted from roots to shoots.

Recent evidence further broadens the significance of PRI by linking root-zone heterogeneity to physiological acclimation, nutrient dynamics, and belowground interactions. In potato, arbuscular mycorrhizal fungi were shown to maintain or improve plant growth and phosphorus and nitrogen uptake under low-phosphorus conditions, combined with DI or PRD, indicating that root-associated biota can partly buffer irrigation-induced nutritional constraints [18]. In barley (*Hordeum vulgare* L.), PRD enhanced low-temperature tolerance by improving gas exchange, reducing relative conductivity, and promoting root antioxidant and metabolic adjustments [19]. Work in alfalfa (*Medicago sativa* L.) further indicates that PRD-driven changes in photosynthesis may involve both stomatal and non-stomatal limitations within the same day, highlighting that the physiological consequences of PRD cannot be explained by stomatal closure alone [20]. In parallel, split-root studies in tomato showed that PRD can regulate stomatal conductance and leaf growth through long-distance signaling [21], while root excision experiments in apple (*Malus domestica* Borkh). demonstrated that roots in drying soil can suppress shoot growth independently of any additional water supply, thereby providing direct support for root-sourced regulation [22]. Moreover, alternation of wet and dry sides has been shown to modify root-to-shoot ABA signalling itself, underscoring the importance of switching frequency and irrigation design for maintaining a sustained PRI effect [23,24]. Together, these findings suggest that PRI may be better understood as a form of spatial stress priming: localized soil drying does not simply reduce water loss but may reorganize signaling, metabolism, and root-associated processes in ways that improve subsequent stress acclimation.

Despite substantial progress, the literature remains fragmented in several important respects. First, many studies still assess PRI primarily in terms of irrigation saving and yield maintenance, while mechanistic integration among hydraulic signaling, hormone crosstalk, nutrient dynamics, root metabolism, and rhizosphere responses remains incomplete. Second, drought-related PRI responses are considerably better characterized than responses under salinity, low temperature, or combined-stress scenarios. Third, it is often difficult to determine how much of the apparent PRI advantage is caused by spatial root-zone heterogeneity itself, and how much simply reflects mild water deficit or reduced total irrigation. This distinction is critical because comparisons between PRI and full irrigation alone cannot separate spatial signaling effects from water-saving effects. Strong inference requires PRI to be compared with deficit-irrigation treatments that receive the same total water amount but are applied uniformly across the whole root zone. Only such matched PRI–DI contrasts can identify whether wet–dry partitioning provides benefits beyond reduced irrigation volume. Finally, although PRI research has expanded substantially, the literature has historically been weighted toward horticultural systems, while mechanistic work in agronomic crops has remained comparatively limited. Against this background, the present review is organized around the proposition that PRI should be understood not only as a water-saving irrigation practice, but also as a signaling-based, spatially structured stress-priming strategy in agricultural crops under abiotic stress. On this basis, the review aims to synthesize the current understanding of the signaling mechanisms underlying PRI, evaluate its effects on physiological and biochemical acclimation across major abiotic stresses, and identify the principal conceptual and experimental gaps that must be resolved before PRI can be translated into a robust framework for climate-resilient crop production.

## 2. Partial Root-Zone Irrigation as a Spatial Root-Zone Strategy

**Figure 1:**
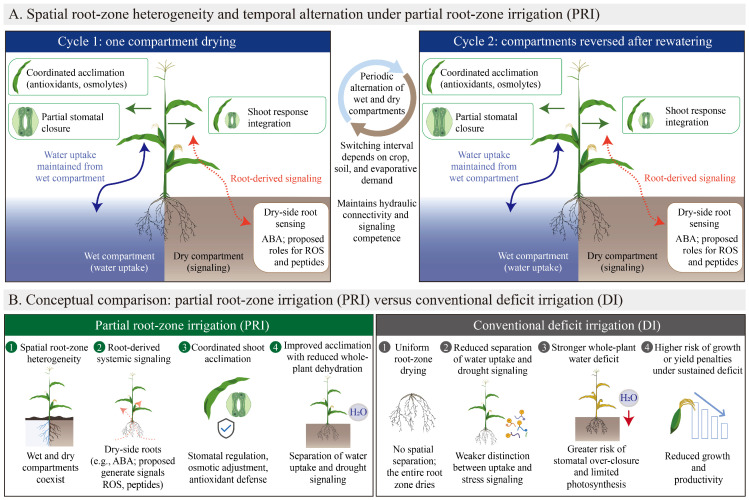
Spatial logic of partial root-zone irrigation under abiotic stress. In partial root-zone irrigation (PRI), root compartments alternate between wet and dry conditions. This allows one part of the root system to take up water, while the other senses localized drying and sends systemic signals. The upper panel shows two irrigation cycles, illustrating how temporal alternation maintains hydraulic connectivity and signaling ability. The lower panel compares PRI to conventional deficit irrigation. PRI creates differences within the root system, helping to coordinate stomatal regulation and physiological acclimation without causing uniform dehydration across the root zone.

PRI is conceptually distinct from conventional deficit irrigation because it is defined not only by the amount of water applied, but by where water is applied within the root zone. In PRI, one part of the root system is maintained in wet soil to sustain water uptake, whereas another part is exposed to drying soil and functions as a source of root-derived drought signaling [25,26]. This spatial arrangement is the central biological feature of the system. As summarized in Table 1 and conceptually illustrated in Figure 1, PRI should therefore be understood as a structured root-zone treatment that generates internal hydraulic and signaling asymmetry, rather than as a simple reduction in irrigation volume. This distinction is fundamental for the rest of the review, because it explains why PRI can produce responses that differ mechanistically from those induced by whole-root-zone deficit irrigation.

### 2.1. Definition of PRI and Distinction from Deficit Irrigation

Partial root-zone irrigation is an irrigation system in which only a fraction of the root system is wetted at any given time, while the remaining fraction is allowed to partially or progressively dry. In its most widely discussed form, PRD, the wet and dry portions are alternated over time according to crop demand, soil drying rate, and irrigation design [25]. Under conventional deficit irrigation (DI), by contrast, the entire root zone receives a uniform reduction in water supply. The key difference is therefore not simply quantitative but structural: DI imposes a relatively even reduction in water availability across the whole root system, whereas PRI imposes within-root-system moisture contrast. Importantly, this does not mean that all downstream responses are unique to PRI. ABA accumulation, stomatal regulation, osmotic adjustment, and antioxidant activation can also occur under conventional DI. The distinction is that PRI reorganizes how these responses are initiated and coordinated by separating drought perception in the drying root compartment from water uptake in the wetted compartment [26]. Thus, PRI should be distinguished from DI not by the exclusive presence of these stress responses, but by their spatial origin, hydraulic context, signal delivery, and coordination under heterogeneous root-zone conditions. Responses such as ABA accumulation, stomatal limitation, osmolyte accumulation, antioxidant activation, and ROS detoxification should be treated as shared water-deficit responses unless PRI–DI comparisons reveal altered timing, magnitude, localization, or physiological outcomes [10]. By contrast, responses more directly attributable to root-zone heterogeneity include simultaneous separation of water uptake and drought perception, dry-side signal generation with wet-side hydraulic support, sap-flow-dependent signal delivery from contrasting root compartments, and renewed signaling capacity after wet–dry alternation [4,8]. However, this structural distinction does not by itself prove that PRI effects are caused by spatial heterogeneity. To attribute an advantage specifically to PRI, the treatment must outperform or mechanistically differ from a DI treatment receiving the same total irrigation amount. Otherwise, improved WUE, reduced transpiration, or yield maintenance may reflect mild deficit exposure rather than a true spatial root-zone effect [25,26]. This distinction is more than terminological. PRI was originally proposed as a biologically informed deficit-irrigation strategy designed to manipulate root-to-shoot signaling and thereby improve agricultural water-use efficiency through endogenous plant regulation [27]. Its theoretical basis lies in the expectation that a modest reduction in stomatal aperture can disproportionately reduce transpirational water loss relative to carbon gain, provided that shoot hydration is not severely compromised [28,29]. PRI attempts to achieve this balance by separating water uptake from drought perception within the same root system: one side remains hydraulically supportive, while the other becomes a localized signaling compartment [25,26]. The conceptual distinctions among conventional irrigation, DI, and PRI are synthesized in Table 1. As shown there, these irrigation strategies differ not only in irrigation amount, but also in the type of heterogeneity imposed, the scale of implementation, the readouts typically measured, and the physiological expectations that arise from each design [30,31]. Conventional irrigation minimizes root-zone contrast; DI creates a temporal deficit across the whole root system; PRI, in contrast, combines spatial heterogeneity with a signaling function that is intrinsic to its design.

### 2.2. Root-Zone Heterogeneity and Alternating Wet–Dry Exposure

The biological relevance of PRI depends on the coexistence of wet and drying root compartments. The wet side maintains water uptake, xylem flow, and shoot hydration, whereas the drying side functions as a source of drought-related signals that influence shoot behavior [32]. However, the dry side must remain sufficiently active to contribute at least some sap flow to the transpiration stream. If drying becomes too severe, roots in the dry compartment may synthesize stronger chemical signals but export less total information to the shoot because their hydraulic contribution declines [33]. PRI is therefore not a simple “half-wet, half-dry” system. It is a dynamic hydraulic-signaling arrangement in which the drying roots must remain dry enough to signal, but not so dry that they become functionally disconnected. This explains why alternation of wet and dry sides is physiologically important rather than merely operational. Prolonged exposure of one root sector to drying soil may reduce radial permeability, promote loss of succulent secondary roots, and progressively isolate that compartment from the transpiration stream. Rewetting and alternation can reverse part of this decline by stimulating renewed root activity and secondary root development, thereby restoring the capacity of that compartment to perceive later drying episodes [34]. In this sense, PRI combines spatial heterogeneity with temporal renewal: spatial contrast generates the signal, whereas periodic switching helps preserve the signaling competence of the root system over repeated drying–rewetting cycles. This logic is captured visually in Figure 1, which presents PRI as a spatial stress-priming strategy rather than merely a reduced-water irrigation method. The figure emphasizes that localized drying is perceived in one root compartment while water uptake is maintained from the wet side, allowing systemic signals to reach the shoot without imposing the same kind of uniform whole-root-zone deficit that characterizes DI. Read together, Figure 1 and Table 1 make the same conceptual point from different angles: the figure highlights the biological process, whereas the table clarifies the design variables and experimental contrasts that define PRI research. Root-zone heterogeneity also determines how soil properties are translated into plant responses. Under PRI, the physiological outcome depends not only on soil water content or matric potential in the drying compartment, but also on how those variables influence root water potential, sap flow partitioning, and the hydraulic contribution of each root sector to the whole plant [25]. Consequently, design variables such as switching frequency, wet:dry root-zone ratio, rooting volume, soil or substrate type, and irrigation delivery method are not minor technical details. They also determine whether PRI can be distinguished experimentally from a mild whole-root-zone deficit [15]. A rigorous PRI design should therefore include at least three contrasts: full irrigation, uniform DI with the same total water input as PRI, and PRI with spatially separated wet and dry compartments. Where possible, studies should also quantify soil moisture distribution, dry-side sap flow, root water potential, xylem ABA or sap-flow-weighted ABA export, stomatal conductance, photosynthesis, and yield or quality. These shared measurements are needed to separate the effects of reduced irrigation volume from those of root-zone spatial heterogeneity [3,17]. They determine whether the imposed treatment functions as a true signaling system, a simple under-irrigation event, or an unstable intermediate condition. This is why a mechanistic interpretation of PRI requires close attention to the scale of implementation, especially when comparing split-root systems, pot experiments, and field studies.

### 2.3. PRI as a Priming Framework Rather than Only a Water-Saving Method

Historically, PRI has often been described as a water-saving irrigation strategy, and that description remains agronomically valid. Early work on grapevine showed that PRD could improve water-use efficiency by up to 50% without major crop reduction [28], while field studies in potato demonstrated 30% irrigation savings and a 59% increase in crop water-use efficiency [29]. Similar feasibility was later reported in tomato systems under both greenhouse and field conditions [30,31]. Yet these outcomes should not obscure the more important conceptual point: PRI does not simply reduce water input; it reorganizes where drought is sensed within the root system and how that information is communicated to the shoot. This signaling-based interpretation is supported by evidence that PRI affects more than just stomatal conductance. In tomato, PRD altered both xylem abscisic acid and cytokinin relations, indicating that PRD alters hormonal balance reaching the shoot rather than triggering a single, isolated ABA response [27]. In apple, excision of roots in drying soil restored shoot growth and stomatal opening without providing any additional water, demonstrating directly that dry-side roots can impose inhibitory control over shoot function through root-sourced signals [35]. Long-distance signaling studies in tomato likewise showed that PRD regulates both stomatal conductance and leaf growth, thereby linking root-zone heterogeneity to developmental and hydraulic responses [36]. These observations collectively suggest that the effect of PRI is not limited to water conservation per se but extends to the regulation of whole-plant function through spatially organized signaling. For this reason, PRI may be understood as a priming framework when its effects exceed those produced by an equivalent uniform deficit. This distinction is essential: if PRI and DI receive the same total irrigation water but produce similar physiological and yield responses, the advantage is more likely attributable to mild deficit irrigation or water-saving. If PRI produces distinct xylem signalling, stomatal regulation, root hydraulic behaviour, or stress-acclimation responses relative to matched DI, then spatial root-zone heterogeneity becomes a more plausible mechanistic explanation. By repeatedly exposing one part of the root system to localized, non-lethal drying while another part remains hydrated, PRI may maintain stress-responsive pathways in a partially activated state without causing catastrophic dehydration. Such repeated, spatially structured exposure is consistent with a priming-like interpretation in which the plant is not merely stressed during irrigation treatment but may acquire enhanced readiness for later abiotic challenges. However, as defined in Section 2.4, PRI should be classified as true stress priming only when experiments demonstrate a prior PRI stimulus, a separable subsequent stress challenge, and improved later performance relative to appropriate controls. Even in lemon, where improvements in water-use efficiency under PRD appeared independent of increased leaf xylem ABA concentration, the response still indicated a broader systems-level adjustment than irrigation saving alone [37]. Accordingly, Table 1 and Figure 1 should be read together: Table 1 defines the conceptual and design distinctions among irrigation strategies, whereas Figure 1 synthesizes the central argument that PRI functions as a spatial stress-priming strategy built on root-zone heterogeneity.

### 2.4. Defining Stress Priming in the Context of PRI

Stress priming is a process in which a prior stimulus, usually moderate and non-lethal, prepares a plant to respond more effectively to a later stress event. In the primed state, the plant does not necessarily maintain a fully activated stress response; rather, it acquires an enhanced capacity to respond faster, more strongly, or more efficiently when subsequently challenged [38]. This definition is consistent with current plant-stress literature, in which priming is distinguished from immediate acclimation by a temporal sequence: a priming stimulus, a post-stimulus interval or memory phase, and an improved response to a later stress exposure [39]. In this sense, priming differs from stress acclimation, which refers to physiological adjustment during an ongoing stress; from hardening, which generally describes increased tolerance after repeated or sustained exposure to a stress, often without requiring a clearly separable later challenge; and from preconditioning, which is a broader term for any prior treatment that improves later performance but may not demonstrate stress memory or enhanced response kinetics [40]. For PRI to be classified strictly as stress priming, at least four criteria should be satisfied. First, localized wet–dry root-zone exposure should act as a non-lethal priming stimulus rather than as damaging stress. Second, the plant should show a measurable physiological, biochemical, molecular, or developmental change after PRI exposure [41]. Third, improved performance should be demonstrated during a later stress challenge relative to an appropriate control, preferably including both full irrigation and matched deficit-irrigation treatments [38]. Fourth, the response should show evidence of enhanced readiness or memory, such as faster stomatal regulation, stronger antioxidant activation, improved maintenance of photosynthesis, altered hormone sensitivity, persistent metabolic or transcriptional marks, or improved recovery after stress [39,40,41]. In the current PRI literature, these criteria are not always fully tested. Therefore, throughout this review, PRI is described as “priming-like” when evidence indicates preparatory acclimation or enhanced stress readiness, and as “stress priming” only where the experimental design supports a prior stimulus followed by improved later stress response [39,40,41].

**Table 1:** Conceptual distinctions and design variables in partial root-zone irrigation research.

Irrigation Design	Water Distribution Pattern	Heterogeneity Type	Typical Implementation	Key Design Variables	Key Readouts Usually Measured	Typical Physiological Expectation	References
Conventional irrigation (full/root-zone irrigation)	Whole root zone wetted; minimal internal contrast	Low spatial heterogeneity; low temporal stress cycling	Full furrow, basin, sprinkler, or full-profile drip irrigation	Irrigation amount; interval; ET replacement	Soil water status; transpiration; biomass; yield; baseline WUE	High stomatal conductance and transpiration; minimal drought signalling; maximal shoot hydration	[25,26]
Deficit irrigation (DI/RDI)	Entire root zone receives less than full crop water requirement	Mainly temporal deficit; little internal spatial contrast	Reduced full-zone furrow or drip irrigation; stage-specific regulated deficit irrigation	Degree of deficit; timing; developmental stage; soil type	Leaf water potential; gas exchange; biomass; yield penalty; WUE	Whole-root-zone drought exposure; reduced transpiration and photosynthesis; limited separation between signalling and water uptake	[25,30,31]
Fixed partial root-zone drying (non-alternating PRI)	One compartment wet, one dry, but position not switched	Strong spatial heterogeneity; weak temporal renewal	Split-root columns; twin pots; divided root systems; mechanistic greenhouse studies	Wet:dry ratio; rooting volume; substrate; duration of drying	Xylem ABA/CK; leaf water status; stomatal conductance; leaf growth; sap flow fractions	Strong dry-side signalling with maintained hydration, but risk of dry-root hydraulic isolation and declining signal export over time	[26,27,35,36]
Alternating partial root-zone irrigation (PRD/CAPRI)	One side wet and one side drying, with wet and dry sides alternated	Coupled spatial and temporal heterogeneity	Split-root pots; alternate furrow irrigation; partial drip; subsurface drip; orchard row-side alternation	Switching frequency; wet:dry ratio; soil/substrate; irrigation method; crop architecture	Moisture asymmetry; dry-side sap flow; xylem ABA; hydraulic conductance; root proliferation; nutrient uptake; WUE; yield/quality	Maintained water uptake from wet roots plus recurrent signalling from dry roots; renewed root activity after rewetting; higher WUE and priming-like acclimation potential	[28,32,33,34,37]

Note: PRI, partial root-zone irrigation; PRD, partial root-zone drying; CAPRI, controlled alternate partial root-zone irrigation; DI, deficit irrigation; RDI, regulated deficit irrigation; WUE, water-use efficiency; ABA, abscisic acid; CK, cytokinin.

## 3. Signaling Basis of PRI

**Figure 2:**
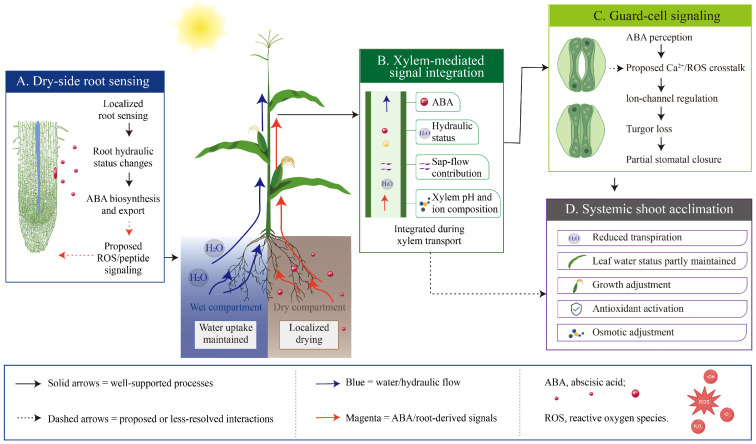
Root-to-shoot signaling architecture under partial root-zone irrigation. Schematic representation of how heterogeneous root-zone moisture under PRI is translated into systemic regulation. Localized drying in one root sector promotes root sensing, ABA biosynthesis, and hydraulic modulation, while the wet sector maintains transpiration stream connectivity. Signal transfer through the xylem integrates hormone concentration, sap-flow contribution, pH, and ion composition, and hydraulic status, thereby influencing stomatal conductance, leaf water relations, and downstream stress responses. Dashed arrows should denote proposed or less-resolved interactions, including crosstalk with ROS-, calcium-, and jasmonate-related signaling pathways.

PRI is hypothesized to operate most specifically when spatial variation in soil moisture is translated into systemic physiological information beyond that caused solely by reduced irrigation volume [35]. This distinction is necessary because many downstream responses discussed in this review, including ABA accumulation, stomatal regulation, osmolyte accumulation, antioxidant activation, and ROS detoxification, are general components of plant responses to water deficit and can also be induced by conventional DI [37]. These responses should therefore not be used alone as evidence for PRI-specific root-zone heterogeneity [39]. Instead, PRI-specific inference requires evidence that heterogeneous wet–dry compartments alter the spatial source, hydraulic delivery, temporal dynamics, or coordination of these responses relative to uniform DI. Under PRI, the key mechanistic question is not whether these responses occur, but whether heterogeneous wet–dry root-zone conditions alter their timing, intensity, hydraulic coupling, or coordination relative to uniform deficit conditions [40]. Once the root system is simultaneously exposed to wet and drying compartments, the plant responds not only to the total amount of water available in the soil, but also to its distribution across the root zone. This is the central mechanistic feature that distinguishes PRI from whole-root-zone deficit irrigation. Under PRI, the drying compartment functions as a localized source of stress information, whereas the wet compartment sustains water uptake and helps stabilize shoot water status [25,26]. In this way, drought-responsive regulation can be activated without necessarily imposing the same degree of generalized dehydration that characterizes uniform soil drying. The principal components of this signaling architecture are summarized in Figure 2.

### 3.1. Root Perception of Heterogeneous Soil Moisture

The initiation of PRI signaling depends on the roots’ capacity to perceive localized soil drying and convert that stimulus into mobile signals. Early work on plants growing in drying soil showed that roots can influence stomatal behavior, leaf expansion, and shoot development before substantial changes in leaf water status become detectable, establishing the root system as an early sensor of stress rather than merely a passive site of water uptake [1,42]. Under PRI, this principle becomes spatially explicit: only part of the root system experiences drying, yet the resulting response is expressed at the whole-plant level. The drying compartment, therefore, assumes a regulatory role that extends beyond its direct contribution to water absorption [43]. This sensing process is not solely governed by soil water content. Its effectiveness depends on how local drying alters root hydraulic status and on whether the drying roots remain sufficiently connected to the transpiration stream to export information to the shoot [44,45]. In sunflowers, root water potential integrated the effects of contrasting substrates on xylem ABA concentration more consistently than soil variables alone, indicating that roots respond to drying through a hydraulic state that reflects, but does not simply replicate, soil water availability [26]. This distinction is important because roots in severely dry soil may still accumulate signaling molecules while losing much of their capacity to export them if their hydraulic contribution to whole-plant flow becomes too small. The efficiency of perception under PRI is therefore inseparable from the temporal dynamics of the treatment. Prolonged drying of one root sector can reduce radial conductivity, diminish the abundance of active secondary roots, and progressively weaken the capacity of that compartment to perceive and transmit renewed drying signals. Alternation of wet and dry sides restores root activity and renews the signaling competence of the previously dry sector [25,34]. Root perception under PRI is thus not static but repeatedly re-established through wet–dry cycling. Spatial asymmetry initiates the signal, whereas temporal alternation maintains the root system’s capacity to continue generating it.

### 3.2. ABA, Hydraulic Signaling, and Stomatal Regulation

Among the signals associated with PRI, abscisic acid (ABA) remains the most extensively characterized. Roots in the drying compartment typically increase ABA biosynthesis, and the hormone is transported through the xylem to the shoot, where it contributes to stomatal regulation and growth restraint [41,42]. However, the significance of ABA under PRI is determined not only by its concentration but also by its delivery. What reaches the shoot depends on both the ABA concentration in xylem sap and the proportion of sap flow contributed by the drying roots [46,47]. Under heterogeneous soil moisture, signal concentration and signal flux are therefore mechanistically distinct. This distinction is central to understanding why PRI does not operate as a simple linear drought response. In sunflower, intensified drying of the dry compartment increased the ABA concentration of xylem sap from that root sector, but at the same time reduced its contribution to total sap flow; once flow from the drying side became too small, total ABA export to the shoot declined despite higher local ABA concentration [26,46]. Effective signaling under PRI, therefore, requires a balance: the drying roots must experience sufficient water limitation to generate a signal, but must remain hydraulically functional enough to transmit that signal. In this sense, PRI is a flow-dependent chemical signaling system.

Hydraulic signaling operates alongside chemical signaling and strongly conditions its effects. Changes in root water potential, leaf water potential, root hydraulic conductance, and xylem transport all shape how ABA is delivered, perceived, and translated into stomatal behavior [42,43]. In grapevine, PRD substantially reduced stomatal conductance even though the increase in foliar ABA was relatively modest compared with severe whole-root-zone drought, suggesting that the hormonal response was effective because it was expressed within an appropriate hydraulic context rather than because of hormone accumulation alone [48]. Similar evidence in lemon showed that improvements in water-use efficiency under PRI could not be explained by enhanced leaf xylem ABA concentration alone [49]. These findings indicate that stomatal regulation under PRI arises from coordinated hydraulic–chemical integration rather than from ABA concentration in isolation. Xylem chemistry provides an additional level of control. In addition to ABA itself, xylem pH and ionic composition can influence ABA partitioning and guard-cell sensitivity, thereby modifying stomatal responses downstream of root drying [50]. Stomatal control under PRI should therefore be viewed not as a direct hormonal reflex, but as the outcome of an integrated signaling environment in which root perception, hydraulic state, sap flow, xylem composition, and shoot sensitivity all interact.

### 3.3. Signal Crosstalk and Systemic Coordination

Although ABA occupies a central position in PRI signaling, the downstream response is broader than an ABA-only model would suggest. One of the clearest indications of this comes from cytokinin dynamics. In tomato, PRD increased xylem ABA concentration and reduced whole-plant transpiration, but also decreased leaf cytokinin status, indicating that localized soil drying alters the hormonal balance reaching the shoot rather than triggering a single isolated ABA response [27]. The physiological outcome of PRI thus depends on hormonal rebalancing at the whole-plant level, mediated through changes in transport, metabolism, and tissue delivery. The signaling network broadens further when PRI is considered in the context of stress acclimation. Moderate, localized drying of part of the root system may activate response pathways that overlap with those involved in cold, osmotic, and oxidative stress. Recent work in barley illustrates this clearly. Under low temperature, PRD improved net photosynthetic rate, stomatal conductance, and photosystem II (Fv/Fm) while reducing relative conductivity; these responses were accompanied by enhanced antioxidant activity, greater glutathione accumulation, and restructuring of the rhizosphere microbiome [19]. Such findings indicate that the signaling consequences of PRI are not limited to water conservation but extend into broader protective and acclimatory processes. For this reason, signal crosstalk under PRI is better interpreted as a coordinated network rather than a single pathway. ABA interacts with hydraulic status, xylem chemistry, and growth-related hormones such as cytokinins; in addition, probable interaction with reactive oxygen species (ROS), calcium, jasmonates, auxin, and other stress-related signaling modules may strengthen the conversion of localized drying into a systemic adaptive response [43,44,51]. Recent hormone-centered stress-regulation frameworks emphasize that auxin biodynamics, including auxin biosynthesis, transport, conjugation, degradation, and Aux/IAA–ARF-mediated signaling, are central to how plants balance growth, root plasticity, and defense under drought, salinity, heat, cold, and combined stress conditions [52]. This is relevant for PRI because localized wet–dry root-zone heterogeneity may alter not only ABA-mediated stomatal regulation, but also auxin-dependent root architecture, lateral root development, nutrient-foraging capacity, and growth–defense tradeoffs. Some components of this network are already well supported, particularly ABA–hydraulic integration and ABA–cytokinin coordination, whereas auxin-mediated regulation of PRI responses remains less directly tested and should currently be interpreted as a plausible extension of broader hormone-centred stress biology rather than as an established PRI-specific pathway [46]. Even so, the broader implication is clear: PRI does not operate through a single isolated signal, but through systemic coordination across multiple signaling layers.

This integrated perspective also helps explain why PRI may behave as a priming-like treatment in some experimental contexts [20]. Repeated, localized, non-lethal drying does not merely impose stress; it may maintain components of the plant’s signaling and acclimation machinery in a partially activated or more responsive state [25]. However, this should be interpreted as priming only when enhanced performance is demonstrated during a later stress challenge, rather than inferred solely from responses measured during the PRI treatment itself. Viewed in this way, the significance of PRI lies not only in improving water use but also in reorganizing how the plant anticipates and coordinates stress responses [34]. Figure 2 summarizes this mechanistic framework by linking heterogeneous root-zone moisture with root sensing, ABA biosynthesis and transport, hydraulic modulation, xylem-mediated regulation, downstream stomatal and leaf responses, and probable crosstalk with broader stress-signaling pathways.

For clarity, the physiological and biochemical responses reviewed below can be grouped into two categories. The first category includes shared water-deficit responses, such as reduced stomatal conductance, ABA accumulation, osmotic adjustment, activation of antioxidants, ROS detoxification, and partial maintenance of membrane stability. These occur widely under DI and other drought treatments. The second category includes responses that are more directly linked to PRI design, including maintenance of water uptake from the wet root compartment, signal generation from the drying compartment, sap-flow-dependent ABA delivery, hydraulic buffering of shoot water status, and temporal renewal of dry-side root signaling after alternation. The central question is therefore whether PRI reorganizes common water-deficit responses through spatial heterogeneity, rather than creating entirely novel stress pathways.

## 4. Physiological and Biochemical Acclimation Under PRI

**Figure 3:**
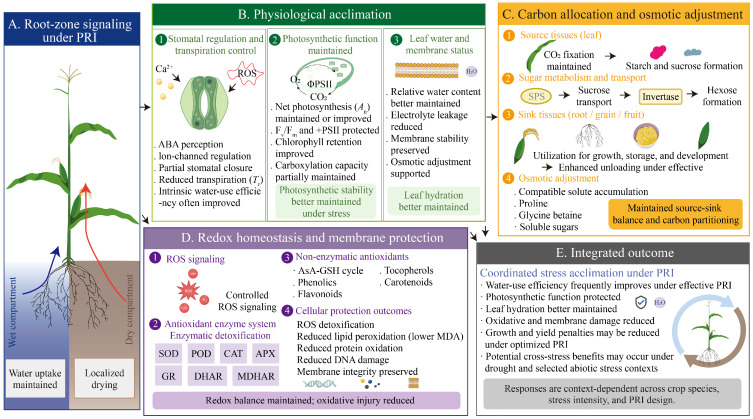
Physiological and biochemical acclimation associated with partial root-zone irrigation. The integrated model shows how PRI-driven root-zone heterogeneity leads to whole-plant acclimation. Root-derived signals and hydraulic buffering affect gas exchange, chloroplast function, and membrane stability. They also influence carbohydrate allocation, osmotic adjustment, antioxidant defense, and redox homeostasis. These responses together can improve stress performance under drought and, in some systems, under low temperature, salinity, or combined-stress conditions. In the figure, solid arrows show well-supported processes. Lighter or dashed arrows indicate pathways that are more inferred or less supported by current evidence.

PRI does not stop at root-to-shoot signaling. Its mechanistic value lies not in producing entirely unique stress responses, but in the possibility that localized root-zone drying coordinates common drought-response modules in a spatially structured way [43]. Many of the responses discussed in this section, including stomatal limitation, osmotic adjustment, antioxidant activation, and membrane protection, are also observed under conventional DI [32]. The PRI-specific interpretation, therefore, depends on whether these responses occur with better preserved shoot hydration, stronger hydraulic buffering, distinct xylem signaling, or improved performance relative to matched DI [17]. Once signaling has been initiated, the response extends across gas exchange, plant water status, membrane stability, carbohydrate partitioning, osmotic adjustment, and redox control. These responses do not operate in isolation; rather, they form an integrated acclimation network through which PRI can sustain function under abiotic stress. The principal pathways discussed in this section are summarized in Table 2, and their integration is outlined in Figure 3.

### 4.1. Photosynthesis, Water Relations, and Membrane Stability

One of the most consistently reported consequences of PRI is moderated stomatal restriction, which can limit transpirational water loss without necessarily imposing an equivalent penalty on carbon gain [10,53]. However, this response is not unique to PRI, because mild uniform deficit irrigation can also reduce stomatal conductance and improve apparent WUE. Therefore, the PRI-specific question is whether spatial wet–dry partitioning produces different stomatal, hydraulic, hormonal, or yield responses from those observed under a uniform DI treatment with the same total water input [22]. In this context, reduced stomatal conductance alone should be treated as a shared deficit response, whereas maintenance of photosynthesis or leaf water status despite partial stomatal restriction provides stronger evidence for a PRI-specific hydraulic-buffering effect [35]. In potato, stomatal conductance was more sensitive to PRD than photosynthesis, allowing crop water-use efficiency to increase by 59% while tuber biomass remained comparable to full irrigation [8]. A similar principle is evident in perennial systems: in lemon, PRD increased crop water-use efficiency by 83% relative to the control, despite the absence of a simple one-to-one relationship between leaf xylem ABA concentration and leaf water-use efficiency [15]. These outcomes highlight the defining physiological logic of PRI: the wet side of the root system provides hydraulic support, whereas the drying side limits excessive water loss through stomatal signaling.

The same pattern becomes even more important when PRI is evaluated under superimposed abiotic stress [54,55]. In barley exposed to low temperature, PRD increased net photosynthetic rate, stomatal conductance, and the maximum quantum efficiency of Fv/Fm by 90.6%, 130%, and 4.6%, respectively, relative to full irrigation under the same cold regime, while relative conductivity declined by 45.2% [19]. These data indicate that PRI can preserve photosynthetic competence and reduce membrane injury under stress, rather than merely restricting gas exchange. In maize under reduced irrigation, PRD combined with biochar likewise improved plant water status, biomass accumulation, and water-use efficiency, even though photosynthesis remained responsive to the degree of water limitation [56]. Thus, the physiological advantage of PRI often lies not in maximizing instantaneous assimilation but in sustaining a more favorable balance between carbon gain, water status, and membrane function over time. Membrane stability is especially informative because it links plant water status, osmotic balance, and oxidative injury [57]. Relative conductivity and allied indicators of electrolyte leakage rise when membranes are destabilized by dehydration, ion toxicity, or oxidative damage. The reduction in relative conductivity under PRI in barley [19], together with improved leaf water relations and hydraulic conductance reported for cotton under salinity when PRD was combined with biochar [58], suggests that PRI can preserve the structural basis on which photosynthesis and metabolism depend. In mechanistic terms, PRI improves stress performance not only by regulating stomata but also by maintaining hydraulic integrity and limiting stress-induced membrane disruption.

### 4.2. Carbon Metabolism, Sugar Turnover, and Osmotic Adjustment

PRI also affects the metabolic fate of assimilated carbon. This is one of the most important yet still incompletely resolved dimensions of the PRI response. Evidence from tomato indicates that PRD can improve fruit biochemical quality traits that are closely linked to carbon partitioning and sugar turnover. Relative to conventional deficit irrigation, alternate PRD significantly increased fruit total soluble solids, glucose, fructose, citric acid, and malic acid, while fruit firmness also increased, and yield remained unchanged [51]. Under elevated atmospheric CO_2_, PRD similarly improved or maintained several quality-related attributes, including total soluble solids, total sugar, total acid, and firmness, while attenuating part of the mineral-quality decline associated with reduced transpiration [56,57]. Although these outcomes are often reported as quality traits, they also imply altered starch breakdown, sucrose turnover, and sink metabolism under PRI.

The direct enzymatic basis of this response remains less well defined than stomatal regulation, but the available evidence strongly suggests a shift in carbon allocation and osmotic metabolism. PRI is consistent with a metabolic pattern in which transient carbon reserves are more effectively mobilized toward soluble sugars and compatible solutes during stress, thereby supporting osmotic adjustment and sink maintenance. In barley, the low-temperature response to PRD was associated with metabolites linked to stress acclimation, including soluble sugars and osmotic substances, as part of the tolerant phenotype [19]. In maize grown under precise PRI, potassium–zinc fertigation under drought increased phosphorus uptake by 53.8%, potassium uptake by 59.2%, proline by 74.4%, and catalase activity by 75.0% under the fixed partial-root treatment, while alternate partial-root treatment increased peroxidase by 81.3% and superoxide dismutase by 82.3% [56]. These changes indicate that PRI can enhance both osmotic and nutritional adjustment, particularly when combined with supportive nutrient management. For this reason, osmotic adjustment should be regarded as an important component of PRI-associated acclimation, but not as a response unique to PRI [4]. Proline, soluble sugars, and related osmolytes commonly accumulate under conventional DI and other drought treatments. The relevant PRI-specific question is whether spatial wet–dry partitioning promotes osmotic adjustment while maintaining greater water uptake, photosynthetic activity, or yield stability than an equivalent uniform deficit [6]. Soluble sugars, proline, and related osmolytes lower cellular osmotic potential, stabilize proteins and membranes, and help preserve hydration during stress. Gas-exchange regulation limits water loss, but osmotic adjustment helps maintain the intracellular environment required for photosynthesis, membrane integrity, and enzyme function. This is why Figure 3 places starch and sucrose metabolism together with osmotic regulation: under PRI, carbohydrate turnover and osmolyte accumulation are functionally linked components of the same adaptive reprogramming.

### 4.3. Redox Homeostasis and Antioxidant Defense

A further frequently reported feature of PRI-associated acclimation is the strengthening of antioxidant capacity and redox balance. However, antioxidant activation is a common response to water deficit and oxidative stress and should not be treated as specific to PRI [15]. What may distinguish PRI is the extent to which antioxidant responses are coordinated with maintained hydraulic function, partial preservation of photosynthesis, and reduced membrane injury under heterogeneous root-zone conditions compared with matched DI [28]. Abiotic stress commonly increases ROS production, and the resulting oxidative pressure can damage membranes, pigments, proteins, and the photosynthetic apparatus [58]. PRI appears to moderate this process by activating antioxidant defense systems and by reshaping the balance between ROS generation and detoxification [59,60]. In barley under low temperature, PRD enhanced root peroxidase and monodehydroascorbate reductase activities while increasing glutathione accumulation in roots [19]. At the same time, the transcriptomic response under PRD was enriched in oxidoreductase, peroxidase, and antioxidant-related pathways, indicating that the redox response was coordinated at both metabolic and transcriptional levels [19]. The glutathione response is especially important because it places PRI within a broader redox-control framework. Under low temperature, PRD increased reduced glutathione concentration in barley roots, and exogenous glutathione further improved Fv/Fm by 23.4% while decreasing relative conductivity by 12.7% [19]. Likewise, inoculation with *Mortierella alpina* increased root and leaf glutathione by 29.1% and 14.7%, respectively, under low temperature, while also decreasing relative conductivity [19]. These findings indicate that PRI does not merely reduce oxidative injury indirectly through improved water status; it also promotes active reprogramming of antioxidant metabolism. Comparable tendencies are reported in maize, where antioxidant responses in both roots and leaves have been linked to PRI [60], and in alternate partial-root irrigation systems where increases in catalase, peroxidase, and superoxide dismutase accompanied improved drought performance [56].

From a mechanistic standpoint, ROS balance under PRI is likely regulated through both direct and indirect pathways. Directly, PRI can enhance antioxidant enzyme activity and redox metabolites such as glutathione. Indirectly, it can reduce oxidative burden by preserving water relations, moderating ion toxicity, sustaining photosynthetic efficiency, and strengthening osmotic balance. Table 2 places antioxidant defense and ROS balance alongside ABA signaling, stomatal regulation, and carbon metabolism because these processes are functionally interdependent rather than sequentially isolated. Overall, the available evidence supports a model in which PRI enhances abiotic stress acclimation through tightly connected physiological and biochemical pathways: stomatal regulation limits water loss, improved hydraulic status supports photosynthetic continuity, carbohydrate turnover and osmolyte accumulation stabilize metabolism, and antioxidant activation preserves redox balance and membrane integrity. Figure 3 integrates these processes into a single scheme of physiological and biochemical reprogramming under PRI.

**Table 2:** Mechanistic pathways through which partial root-zone irrigation enhances abiotic stress acclimation.

Pathway	Representative Markers	Expected Response Under PRI	Functional Consequence Under Stress	Level of Evidence/Key References
ABA signaling	Root/xylem ABA, sap-flow-weighted ABA export, leaf ABA	Generally enhanced signaling from the drying compartment, but strongly constrained by sap flow and hydraulic connectivity. ABA accumulation itself is not unique to PRI; the PRI-specific feature is dry-side signal generation combined with wet-side hydraulic support and sap-flow-dependent ABA delivery.	Earlier stomatal regulation and growth restraint without severe shoot dehydration. PRI-specific inference requires evidence that ABA delivery or timing differs from matched DI.	Strong [25,26,29,37]
Stomatal regulation	gs, transpiration, intrinsic WUE, stomatal traits	gs usually declines more than net photosynthesis; intrinsic WUE often rises. Because stomatal closure also occurs under conventional DI, reduced gs alone should be treated as a shared water-deficit response rather than a PRI-specific mechanism.	Reduced water loss with partial maintenance of carbon gain. Stronger PRI-specific evidence comes from maintained photosynthesis, leaf water status, or yield under the same total irrigation as DI.	Strong [29,32,37,56]
Leaf water status	Leaf water potential, relative water content, hydraulic conductance, leaf water content	Hydraulic status is buffered more effectively than under uniform whole-root-zone deficit. This pathway is more directly linked to PRI when wet-side water uptake maintains shoot hydration while the dry side generates stress signals.	Delayed hydraulic failure and sustained leaf function under stress.	Strong [29,37,58,59]
Photosynthetic performance	An, Fv/Fm, chlorophyll content, fluorescence traits	Often maintained better than expected from stomatal restriction; photochemical efficiency may improve under stress. Photosynthetic maintenance is not exclusive to PRI, but it becomes mechanistically informative when preserved despite partial stomatal limitation and reduced irrigation.	Sustained carbon assimilation and protection of photosystem function.	Strong [19,54,56]
Membrane stability	Relative conductivity, electrolyte leakage, membrane permeability, MDA	Stress-induced membrane injury generally declines under effective PRI treatments. Membrane protection can also occur under mild DI; PRI-specific interpretation depends on whether protection is stronger under spatial wet-dry partitioning than under equivalent uniform deficit.	Preservation of cellular integrity and reduced stress damage.	Strong [52,58,59]
Starch metabolism	Transient starch, starch remobilization, amylolytic activity	Likely enhanced remobilization toward stress-responsive soluble carbon pools; direct enzyme-level evidence remains limited. Current evidence is suggestive rather than PRI-specific, because carbon remobilization is also a general acclimation response under stress.	Supports energy buffering and downstream osmotic adjustment.	Emerging [52,53,54]
Sucrose metabolism	Sucrose, glucose, fructose, invertase, sucrose-cleaving activity	Greater soluble-sugar accumulation and altered sink metabolism are commonly inferred under PRD. This response should be interpreted cautiously unless matched PRI-DI comparisons show different sugar partitioning or sink activity.	Improved sink strength, quality-related carbon partitioning, and osmotic buffering.	Moderate [53,54,55]
Osmolyte accumulation	Proline, soluble sugars, osmotic potential, compatible solutes	Compatible solutes generally increase under stress-associated PRI treatments. Osmolyte accumulation is a shared water-deficit response and should not be presented as unique to PRI.	Improved turgor maintenance, protein stabilization, and membrane protection. PRI-specific relevance depends on whether osmotic adjustment occurs together with better maintained water uptake, photosynthesis, or yield than under DI.	Moderate–strong [52,57]
Antioxidant enzymes	CAT, POX, APX, SOD, GR, MDHAR, DHAR	Antioxidant capacity is frequently enhanced, although the pattern of individual enzymes varies with crop and stress context. Antioxidant activation is also common under DI and other abiotic stresses; under PRI, the key question is whether it is better coordinated with hydraulic buffering and membrane protection.	Greater ROS detoxification and reduced oxidative injury.	Strong [52,56,61]
ROS balance	H_2_O_2_, O_2_^•−^, GSH, GSSG, AsA–GSH cycle activity	Lower oxidative burden and more efficient scavenging under PRI. ROS detoxification is not PRI-specific; it should be interpreted as part of a broader stress-acclimation module unless PRI-DI comparisons show altered redox timing, magnitude, or tissue localization.	Redox homeostasis and protection of membranes and photosynthetic machinery.	Strong [52,57,62]

Note: ABA, abscisic acid; WUE, water-use efficiency; MDA, malondialdehyde; CAT, catalase; POX, peroxidase; APX, ascorbate peroxidase; SOD, superoxide dismutase; GR, glutathione reductase; MDHAR, monodehydroascorbate reductase; DHAR, dehydroascorbate reductase; GSH, reduced glutathione; GSSG, oxidized glutathione. Several pathways listed here, including ABA accumulation, stomatal regulation, osmolyte accumulation, antioxidant activation, ROS detoxification, and membrane protection, are not unique to PRI and also occur under conventional deficit irrigation. They are included because PRI may modify their spatial origin, timing, hydraulic coupling, signal delivery, and coordination by maintaining water uptake from the wetted root compartment while generating stress signals from the drying compartment. Therefore, PRI-specific inference requires matched comparisons with conventional DI treatments receiving the same total irrigation volume. Evidence is strongest when PRI produces different timing, magnitude, tissue origin, hydraulic context, or agronomic consequences relative to matched DI, rather than simply showing that common drought-response pathways are activated.

## 5. PRI Across Major Abiotic Stresses

PRI was developed in the context of drought, and drought remains the stress condition for which the evidence base is strongest [25]. Evidence from other abiotic stresses suggests that PRI may also influence plant performance beyond water deficit alone, but the strength of support varies substantially among stress types [52]. When one part of the root system dries while another remains hydraulically functional, PRI can generate a signaling environment that modifies water use, gas exchange, and stress-associated acclimation [62]. However, the extent to which this framework applies to low temperature, salinity, heat, or combined stresses depends on the availability of direct experimental evidence, which remains uneven across these contexts. The comparative patterns discussed in this section are synthesized in Table 3.

### 5.1. Drought and Low-Temperature Stress

Drought remains the strongest evidence base for PRI because the treatment itself is built on localized soil drying. Under drought, the dominant PRI-triggered process is the generation of root-derived stress signals from the drying compartment while water uptake is maintained from the wetted side. This arrangement frequently produces a disproportionate decline in stomatal conductance relative to photosynthesis, thereby improving intrinsic or crop-scale water-use efficiency without forcing the same degree of generalized dehydration associated with uniform whole-root-zone deficit [63]. In field-grown potato, PRD maintained tuber biomass similar to full irrigation while saving about 30% irrigation water and increasing crop water-use efficiency by 59% [64]. In grapevine, PRD improved water-use efficiency by up to 50% without significant crop reduction [28]. The broader applicability of this pattern is reinforced by pear-tree studies summarized in the review, where alternative PRI reduced total water use by 12% and irrigation input by 23%, whereas fixed PRI reduced water use by 28% and irrigation input by 52%; in both cases, fruit number and yield per tree were not decreased [65]. Likewise, field studies on maize using alternate furrow irrigation reported that high grain yields could be achieved with up to 50% less irrigation water than with conventional furrow irrigation [25,62]. Overall, these results strongly suggest that drought is the stress context where PRI most consistently improves hydraulic economy and water productivity. However, the size of PRI’s advantage over mild water deficit varies across studies. It can only be estimated from experiments that have a uniform DI comparator using the same total irrigation. When such comparators are missing, reported gains should be seen as benefits of reduced-irrigation strategies in general, not as proof of spatial heterogeneity.

Low temperature provides a mechanistically interesting, but still relatively limited, test of whether PRI-associated signaling can contribute to tolerance under a non-water stress. In barley, low temperature under full irrigation reduced net photosynthetic rate, stomatal conductance, and the maximum quantum efficiency of Fv/Fm by 77.5%, 88.5%, and 6.8%, respectively, while relative conductivity increased by 78.3%, indicating severe physiological disruption [19]. Under the same cold regime, PRD increased the net photosynthetic rate by 90.6%, stomatal conductance by 130%, and Fv/Fm by 4.6%, while reducing relative conductivity by 45.2% compared with fully irrigated plants [66,67]. Importantly, these responses were accompanied by higher root antioxidant activity, increased glutathione accumulation, differential enrichment of genes associated with glutathione and fatty acid metabolism, and the recruitment of rhizosphere core microorganisms, such as *Sphingobium* and *Mortierella* [19]. The barley work is especially valuable because it links the physiological outcome directly to stress-overlap biology: drought and cold are known to share regulatory components involving ABA, calcium-mediated signaling, redox homeostasis, and osmotic adjustment [68,69,70,71,72]. Accordingly, PRI under low temperature may be interpreted as more than an irrigation treatment applied during cold exposure; in barley, it appears to create a signaling and acclimation context that is consistent with cross-stress tolerance. However, this interpretation should remain crop- and context-specific until comparable evidence is available from additional species and factorial stress designs. Even so, compared with drought, direct low-temperature evidence remains limited to relatively few crop systems, and the present confidence level is therefore best described as moderate rather than high.

### 5.2. Salinity and Heat Stress

Salinity represents a second major context in which PRI appears mechanistically relevant. Here, the key issue is not water deficit alone but the joint disruption of hydraulic function, ion homeostasis, and oxidative balance. Evidence from cotton shows that PRI can moderate these effects, particularly when combined with amendments that improve the rhizosphere chemical environment. In one study, wheat-straw biochar had a greater Na^+^ adsorption capacity than softwood biochar (55.20 versus 47.38 mg g^−1^), and, when combined with PRD, it increased the K^+^/Na^+^ ratio, root volume, root surface area density, and water-use efficiency under salt stress [59]. In a complementary experiment, PRD under salinity increased stomatal density, maximum stomatal conductance, and intrinsic water-use efficiency while decreasing stomatal conductance itself, and the combined wheat-straw-biochar plus PRD treatment produced the greatest improvement in leaf water relations and hydraulic conductance [58]. These findings indicate that, under salinity, PRI acts through mechanisms beyond transpirational restraint: it also modifies ion balance, root morphology, and hydraulic integrity. The current confidence level for salinity can therefore be considered moderate, especially in systems where PRI is integrated with soil or nutrient amendments.

The evidence base for heat stress is substantially less mature than that for drought or salinity. Direct factorial studies in which PRI is imposed under well-defined thermal stress, while soil water supply is independently controlled, remain uncommon [50]. Much of the available evidence comes from semiarid or high-vapour-pressure-deficit production environments, where high temperatures, atmospheric drought, and soil water limitation co-occur [57]. Therefore, current heat-related PRI evidence should be interpreted cautiously. It is more accurate to describe this literature as evidence of PRI performance under heat-associated evaporative demand rather than as definitive evidence of heat-only tolerance [60]. In lemon orchards, PRD increased crop water-use efficiency by 83% compared with the control without significantly reducing yield, despite the fact that this gain could not be explained by leaf xylem ABA alone [42]. Viticultural studies in hot semiarid regions provide similar indications. A three-year field study on grapes reported that PRD improved yield by 43% and water-use efficiency by 40% relative to conventional irrigation in the final year of the experiment, although effects were weak or absent in the first two years [62,67]. Other grape studies concluded that PRD maintained or improved fruit composition while reducing vegetative vigor and irrigation demand [25,68].

Mechanistically, heat-only PRI effects would be expected to differ from drought-only PRI effects because heat stress primarily disrupts leaf energy balance, membrane stability, photosystem function, reproductive development, and protein homeostasis [24]. If PRI improves performance under true thermal stress, the most plausible mechanisms would include partial stomatal regulation that limits excessive transpiration while still allowing evaporative cooling, maintenance of leaf water status and canopy temperature control through the wetted root compartment, protection of photosystem II activity, stabilization of membranes, and activation of antioxidant and heat-protective metabolism [18,19,20]. In addition, PRI may influence aquaporin activity, root hydraulic conductance, ABA-mediated stomatal sensitivity, osmolyte accumulation, and heat-shock-protein-related pathways, although direct evidence for these mechanisms under heat-only PRI treatments remains limited. Thus, a heat-specific PRI mechanism should be demonstrated using endpoints such as canopy temperature, leaf energy balance, Fv/Fm, electrolyte leakage, membrane lipid peroxidation, antioxidant enzymes, heat-shock protein expression, aquaporin expression, pollen viability, flowering or fruit-set stability, and yield under factorial heat × irrigation designs [34]. These data suggest that PRI can maintain water-use efficiency and crop performance in environments with heat-associated evaporative pressure. However, they do not yet establish that PRI directly improves heat-stress tolerance independently of water-saving, vapor-pressure-deficit, or drought-avoidance effects [15]. For this reason, evidence for heat-related PRI responses should be classified as low to emerging. Mechanistic claims about heat tolerance should remain provisional until factorial heat × irrigation studies with appropriate DI comparators test whether PRI improves thermal tolerance through heat-specific traits, such as canopy cooling, PSII stability, membrane protection, reproductive heat tolerance, antioxidant regulation, aquaporin-mediated hydraulic adjustment, or heat-shock-protein responses [65].

### 5.3. Combined Stresses and Cross-Tolerance

A conceptually important, but still developing, frontier in PRI research concerns combined stresses and possible cross-tolerance [8,9,10]. Because PRI repeatedly imposes localized, non-lethal drying while preserving water uptake elsewhere, it may keep some stress-response pathways partially activated. At present, however, this should be treated as a plausible working hypothesis rather than an established general mechanism [32]. This provides a biologically plausible basis for cross-tolerance, especially where different abiotic stresses share signaling components. The barley low-temperature study is particularly informative in this regard because it explicitly frames PRI-mediated cold tolerance in relation to drought-type signaling through ABA, calcium-mediated signaling, redox homeostasis, and osmotic regulation [52,71,72]. The same study further suggested that glutathione- and oleamide-associated responses under low temperature may also be relevant under drought stress, indicating possible overlap among downstream protective processes [52]. However, because the causal direction among metabolite changes, microbial shifts, and stress protection remains unresolved, these findings should not be interpreted as proof of a unified microbiome-mediated protective network. Rather, they support the more cautious interpretation that PRI may function as a priming-like treatment in some systems by influencing metabolic and antioxidant readiness, while the contribution of microbial restructuring remains to be validated experimentally.

Evidence for combined stress is emerging from crop systems exposed to simultaneous resource and environmental constraints. In tomato, under elevated atmospheric CO_2_ and reduced irrigation, PRD improved water-use efficiency and several fruit-quality variables, while moderating part of the decline in mineral nutrition associated with reduced transpiration under CO_2_ enrichment [54,55]. This outcome is important because it shows that PRI can remain physiologically advantageous even when drought is embedded within a broader climate-change context rather than acting alone. Likewise, cotton studies combining salinity with water limitation indicate that PRI, particularly when paired with biochar, can improve hydraulic function, ion homeostasis, and stress tolerance under multiple simultaneous constraints [58,59]. At the same time, the available evidence shows that cross-tolerance is neither universal nor cost-free. Under PRD gradients in alfalfa, water productivity and irrigation water productivity shifted in opposite directions depending on the degree of water deficit: severe water deficit maximized irrigation water productivity, whereas over-irrigation produced the highest hay yield and the highest overall water productivity [73]. This emphasizes that the agronomic value of PRI under combined or interacting stresses depends on the balance between stress moderation and growth limitation, not on signaling alone.

Taken together, the available literature suggests that PRI has a robust, conserved function across stress environments: it improves the coordination of plant responses under constraints. Under drought, this is expressed mainly as hydraulic economy and stomatal restraint; under low temperature, as preservation of photosynthetic competence, membrane integrity, and redox balance; under salinity, as tighter coupling among hydraulic status, ion regulation, and root function; and under hot evaporative conditions, as improved water-use efficiency with variable effects on yield and quality. What remains unresolved is the degree to which these outcomes arise from the same underlying signaling architecture and how far PRI can be generalized across fully factorial multi-stress environments. This is the rationale for Table 3, which distinguishes the conserved core of the PRI response from effects that are likely to be stress-specific or supported only by emerging evidence.

**Table 3:** Conserved and stress-specific effects of partial root-zone irrigation under major abiotic stresses.

Stress Type	Main PRI-Triggered Process	Dominant Physiological Outcome	Dominant Biochemical Outcome	Crop Examples	Current Confidence Level	References
Drought	Localized soil drying generates root-to-shoot signaling while the wet side maintains water uptake	Lower plant water use, partial stomatal closure, improved WUE, often limited yield penalty; e.g., potato saved ~30% irrigation water and increased crop WUE by ~59%, while grapevine improved WUE by up to 50%.	Dominated by ABA-linked signaling, better osmotic adjustment, and in some systems improved nutrient use.	Potato, grapevine, pear, maize, cotton	High	[25,28,29,32,62,64,65,66]
Low temperature	PRI recruits drought-type signaling and root metabolic reprogramming under cold exposure	In barley, PRD raised An by 90.6%, Gs by 130%, and Fv/Fm by 4.6%, while reducing relative conductivity by 45.2% under low temperature	Enhanced glutathione metabolism, antioxidant activation, and rhizosphere restructuring	Barley; conceptually extensible to cold-sensitive cereals	Moderate	[52,71,72]
Salinity	Hydraulic moderation plus improved ion balance and root function, especially with co-amendments	Improved root morphology, hydraulic conductance, WUE, and K^+^/Na^+^ relations in cotton.	Reduced Na^+^ burden and improved ion homeostasis; stronger ROS buffering when PRD is combined with biochar.	Cotton	Moderate	[19,58,59]
Heat stress/heat-associated evaporative demand	PRI may moderate transpirational and hydraulic costs under high atmospheric demand, but heat-specific signaling remains poorly resolved because most evidence comes from semiarid or high-VPD field environments rather than controlled heat-only experiments.	Improved WUE and, in some cases, maintained yield or quality under hot semiarid or high-VPD conditions; e.g., lemon showed higher WUE under PRD, and grapevine responses improved under suitable wet–dry cycling. However, these outcomes should be interpreted as heat-associated evaporative-demand responses, not definitive proof of heat-only tolerance.	Possible mechanisms include canopy cooling through maintained wet-side water uptake, partial stomatal regulation that balances water saving with evaporative cooling, protection of PSII activity, membrane stabilization, antioxidant activation, aquaporin-mediated hydraulic adjustment, heat-shock-protein responses, and reproductive protection through pollen viability or fruit-set stability. Direct biochemical evidence under heat-only PRI remains limited.	Lemon, grapevine	Low to emerging; heat, VPD, atmospheric drought, and soil water limitation often co-occur in available field studies, and factorial heat × irrigation experiments with full irrigation, matched DI, and PRI remain scarce.	[28,37,67,68]
Combined stress	Repeated localized drying may pre-condition shared drought/cold/salinity pathways and improve stress coordination	Improved WUE and partial buffering of stress damage, but direction and magnitude remain context dependent.	Shared roles for ABA, redox regulation, osmotic adjustment, ion regulation, and metabolite reprogramming are probable.	Barley under cold with drought-type signaling overlap; tomato under eCO_2_ + reduced irrigation; cotton under salinity + water limitation	Emerging to moderate	[46,48,49,52,53]

Abbreviations: PRI, partial root-zone irrigation; PRD, partial root-zone drying; WUE, water-use efficiency; eCO_2_, elevated atmospheric CO_2_.

## 6. Root-Centered Regulation: Omics, Root Function, and Rhizosphere Responses

**Figure 4:**
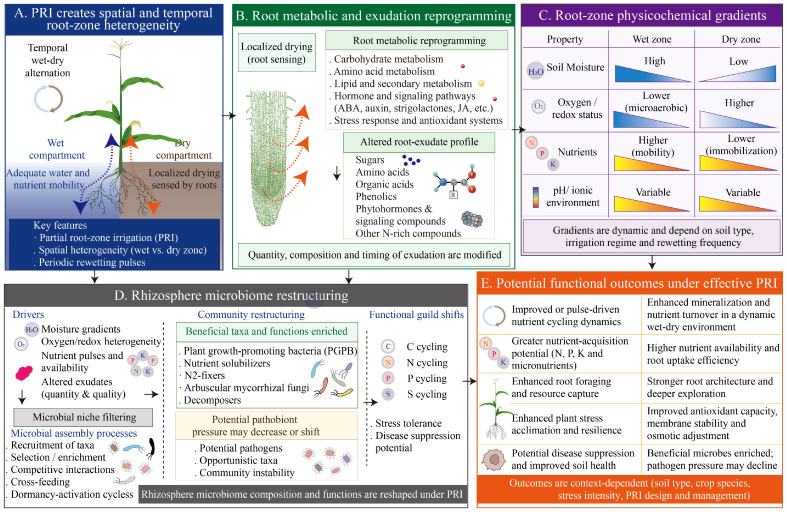
Root–rhizosphere regulation under partial root-zone irrigation. Conceptual model of belowground responses to PRI. Alternating wet–dry compartments alter root metabolism and exudation patterns. They also influence root-zone physicochemical gradients. These changes can reshape rhizosphere microbial assembly and nutrient turnover. The figure highlights a proposed feedback framework that links root reprogramming, rhizosphere restructuring, and plant stress resilience. Since causal direction is often unresolved, visually distinguish supported relationships from hypothesized feedback loops.

PRI is most often discussed in relation to stomatal conductance, transpiration, and water-use efficiency. Yet some of its most distinctive consequences appear to arise belowground [12]. Localized wet–dry alternation appears to modify not only the hydraulic state of the root system, but also several biological features of the root–soil interface. Reported responses include changes in root metabolism, root architecture, nutrient-acquisition traits, root exudation potential, and rhizosphere assembly [24]. However, the evidential strength is not equal across these processes: root hydraulic, architectural, and metabolic responses are better supported than direct causal links between PRI, exudate chemistry, and microbiome assembly [30]. Such processes are especially important because they provide a mechanistic basis for understanding why PRI can generate responses that extend beyond short-term water-saving effects. Importantly, these belowground responses should not be interpreted as a proven linear pathway in which root metabolites necessarily recruit specific microbial taxa [36]. Rather, the current evidence supports a more conservative model in which PRI creates a shared wet–dry physicochemical environment that can simultaneously alter root metabolism, exudation potential, root growth, and microbial community structure, with causal direction still unresolved. In that sense, the root system under PRI should be regarded not merely as a source of signals delivered to the shoot, but also as an active site of biochemical reprogramming and ecological regulation. The principal relationships among these processes are summarized in Figure 4.

### 6.1. Root Metabolic and Molecular Reprogramming Under PRI

The strongest direct evidence for root-centered molecular reprogramming under PRI currently comes from omics-based work in barley exposed to low temperature. In that system, PRD altered not only shoot physiology but also the metabolic and transcriptional state of roots. Metabolomics analysis detected 1169 root compounds, of which 78 differed significantly across the irrigation–temperature contrasts [19]. Within the low-temperature comparison between full irrigation and PRD, seven specific metabolites changed significantly, and five were upregulated under PRD, with particularly strong enrichment of reduced glutathione and 9-octadecenamide [74]. These data are important because they suggest selective metabolic redirection rather than only generalized accumulation of stress products. In other words, PRI may shift root metabolism toward compounds with plausible protective or signaling functions. Nevertheless, this interpretation remains based on a limited number of metabolomic and transcriptomic datasets and requires validation across additional crops, stress combinations, and field-relevant PRI designs [52]. Transcriptomic evidence from the same study reinforces this interpretation. However, these root omics results should also be interpreted in light of recent high-resolution stress-biology frameworks [75]. Bulk metabolomic and transcriptomic analyses are powerful for identifying PRI-associated pathways, but they average signals across multiple root tissues and cell types. Recent advances in single-cell/nucleus RNA sequencing and spatial transcriptomics show that plant stress responses are often cell-type-specific and spatially organized, with distinct transcriptional programs occurring in particular root, vascular, epidermal, cortical, or meristematic cell populations [75]. This is especially relevant for PRI because wet–dry root-zone heterogeneity is itself spatially structured. A bulk root transcriptome may therefore obscure whether PRI-responsive pathways arise from drying-side epidermal and cortical cells, vascular tissues involved in xylem signaling, meristematic zones associated with root plasticity, or cells interacting with rhizosphere microbes [57]. At low temperature, PRD induced a distinct gene-expression profile relative to full irrigation, with enrichment in oxidoreductase and peroxidase activities, antioxidant-associated pathways, and specific changes in glutathione-related metabolism. These findings should also be considered alongside recent evidence that auxin signaling is a major regulator of stress-responsive root development and growth-defense balance [52]. Auxin pathways regulate root architecture, lateral root formation, stress-related gene expression, and interactions with ABA, jasmonic acid, salicylic acid, ethylene, and ROS signaling. Therefore, PRI-induced root architectural plasticity and metabolic reprogramming may involve not only ABA- and redox-centered mechanisms, but also auxin-dependent adjustment of root growth, stress perception, and resource acquisition [52]. Notably, PepA and ACACA were upregulated, while GST and GPX were downregulated, a pattern consistent with enhanced glutathione synthesis and altered fatty-acid or oleamide-related metabolism. This is mechanistically relevant because it connects metabolite accumulation to pathway-level transcriptional control rather than treating metabolite change as a purely descriptive endpoint. The evidence therefore supports the view that PRI can induce a distinct root biochemical state, rather than merely a transient drought signal.

The biological plausibility of this root-centered reprogramming is strengthened by functional assays. In barley, exogenous glutathione and 9-octadecenamide increased Fv/Fm under low temperature by 23.4% and 22.9%, respectively, while reducing relative conductivity by 12.7% and 19.2% [65]. These experiments do not establish that PRI acts exclusively through these metabolites, but they do show that metabolites enriched in roots under PRD are themselves capable of reproducing part of the stress-protective phenotype. This is a critical distinction. A metabolite that is merely associated with the treatment may not have mechanistic importance; a metabolite that can partially restore stress tolerance when supplied exogenously has a stronger claim to functional relevance. However, this functional relevance applies primarily to the plant stress-tolerance phenotype and, by itself, does not demonstrate that the same metabolite causally structures the rhizosphere microbiome [69]. Thus, glutathione and 9-octadecenamide should be interpreted as candidate protective metabolites under PRD, not yet as confirmed microbial recruitment signals. From a review perspective, this makes the barley dataset unusually valuable because it moves the field beyond purely descriptive omics toward functional inference for selected metabolites, while still falling short of establishing metabolite-driven microbiome assembly [70]. At the same time, the next conceptual advance will require moving from bulk-root signatures to cell-type-resolved and spatially resolved interpretation. For example, glutathione-related reprogramming may have different implications if it is concentrated in vascular tissues involved in long-distance signaling, root epidermal or cortical cells exposed to drying soil, meristematic regions controlling root architectural plasticity, or rhizosphere-facing cells involved in exudation [74]. Integrating PRI experiments with single-cell/nucleus RNA sequencing, spatial transcriptomics, and targeted metabolite imaging would help determine which root cell types perceive wet–dry heterogeneity, which transmit systemic signals, and which participate in metabolic or rhizosphere responses [25,34]. At the same time, PRI-induced root regulation is not limited to omics signatures. Structural reorganization of the root system is another recurring feature. In tomato grown under elevated atmospheric CO_2_, PRI increased root length, root surface area, root volume, root dry weight, and root-to-shoot ratio relative to well-watered conditions, while also maintaining or improving nutrient status and water-use efficiency under reduced irrigation [76]. The authors interpreted PRI as promoting a more exploratory root system, with greater capacity for water and nutrient acquisition under freshwater limitation. Similar conclusions were drawn from earlier studies in tomato, maize, grapevine, and rapeseed (*Brassica napus* L.), in which PRD enhanced root growth, stimulated secondary root development, improved soil–root hydraulic conductivity, and shifted biomass allocation toward the root system [69,70,71]. These findings are important because they show that root-centered regulation under PRI is expressed not only at the metabolite or gene level, but also in the architecture through which roots interact with soil resources.

This structural plasticity has direct mechanistic implications. Root proliferation alters the effective contact area between the plant and the soil matrix, modifies nutrient interception, and changes the spatial pattern of rhizodeposition. In grapevine, partial drying of the root zone altered root development patterns and promoted renewed growth following alternation and rewetting [71]. In maize, PRI increased soil–root hydraulic conductivity, demonstrating that the root system can be functionally reconfigured under alternating wet–dry supply [77]. In tomato, PRD-induced enhancement of root growth has likewise been linked with changes in biomass allocation and with improved resource capture under heterogeneous moisture supply [70]. Collectively, these observations suggest that PRI can alter both the metabolic state and the foraging structure of the root system, thereby influencing how the plant explores and exploits the soil environment. A closely related dimension is nutrient acquisition. Repeated drying–rewetting cycles can stimulate organic matter turnover and nutrient release, and PRI appears able to exploit that pulse-driven environment. In maize under alternate partial root-zone drying irrigation (APRI), the combination of APRI with wheat-straw biochar increased microbial activity by 26.8–51.2%, soil N availability by 4.8–13.2%, root growth by 7.4–22.7%, plant N uptake by 7.0–17.8%, total dry biomass by 13.5%, water-use efficiency by 26.7%, and nitrogen-use efficiency by 10.3% [67]. Although the biochar treatment complicates attribution to APRI alone, the broader inference is robust: PRI-type wet–dry cycling can interact with root function and soil biological activity to shift the plant toward a more acquisitive belowground phenotype. Thus, root-centered regulation under PRI is best understood as multidimensional, involving molecular reprogramming, architectural change, and simultaneously plastic resource-capture.

### 6.2. Rhizosphere and Microbiome Responses

The rhizosphere represents one of the strongest opportunities to move PRI research beyond classical leaf physiology. Dry–wet alternation changes oxygen availability, solute diffusion, microsite redox conditions, nutrient mobility, and the temporal pattern of substrate release from roots. PRI therefore creates a rhizosphere that is heterogeneous both spatially and temporally [78]. This heterogeneity is biologically meaningful because rhizosphere microbial communities are highly responsive to local physicochemical conditions and to the quantity and composition of root-derived carbon. Recent evidence indicates that PRI can be associated with changes in rhizosphere assembly, although these patterns should not yet be interpreted as proof of microbiome-mediated PRI responses. In barley, PRD under low temperature did not significantly alter overall bacterial or fungal diversity, but it did alter community composition and network structure. At present, such observations are best treated as evidence of rhizosphere restructuring associated with PRI, not as direct evidence that microbial shifts drive plant acclimation [65]. This distinction is mechanistically important. Diversity metrics may remain stable while functionally important taxa and interactions are reconfigured. Under PRD plus low temperature, the barley rhizosphere remained dominated by *Actinobacteria* (33.6–37.4%), *Proteobacteria* (27.1–31.0%), and *Acidobacteria* (16.3–17.1%), but PRD significantly increased the relative abundance of *Proteobacteria* [65]. Weighted gene correlation network analysis partitioned the bacterial community into 16 modules, with the blue module showing the strongest correlation with the low-temperature PRD treatment (R = 0.68, *p* < 0.05) and positive association with glutathione and 9-octadecenamide [65]. The core taxa of this module included *Myxococcus*, *Gemmatimonas*, *Caenimonas*, and *Sphingobium*. In the fungal community, Mortierellomycota increased under PRD, and network analysis identified a black module strongly associated with the treatment (R = 0.57, *p* < 0.05), with *Mortierella* as the inferred core genus [65].

The relevance of these findings lies in the possibility that PRI influences not only which taxa are present but also which microbial functions are favored. *Sphingobium* has been linked with enhanced root antioxidant performance under stress [65], and *Mortierella* is widely recognized as a metabolically versatile fungal group with potential roles in fatty-acid metabolism, nutrient turnover, and stress-responsive interactions. In the barley system, these microbial associations were statistically connected with metabolites implicated in stress protection. However, this evidence should be interpreted as correlation, not causation [18,79]. The co-occurrence of PRD-enriched metabolites with specific bacterial and fungal modules indicates metabolic coherence between root responses and rhizosphere restructuring, but it does not show that the metabolites directly recruited those microbes, that the microbes caused the metabolite shifts, or that microbial restructuring drove the improved plant phenotype [51]. Equally plausible is that microbial shifts occurred as downstream consequences of PRI-altered root physiology, exudation patterns, oxygen availability, nutrient fluxes, or microsite redox conditions [44,45,46,47,48]. Thus, the barley example provides strong associative evidence for coordinated root–microbiome responses under PRD, while leaving the mechanistic sequence unresolved. This conclusion is strengthened when the barley results are placed in a broader rhizosphere context. Root exudates are widely recognized as important regulators of rhizosphere assembly and function [73,74]. Through sugars, amino acids, organic acids, phenolics, and signaling molecules, roots can shape the microbial habitat immediately surrounding them, thereby influencing nutrient cycling, pathogen suppression, and stress buffering. Nevertheless, in the specific context of PRI, direct evidence connecting PRD-induced metabolite changes to microbial recruitment remains limited. Therefore, general knowledge of exudate–microbiome interactions should be used to frame plausible mechanisms, not to infer causality from correlation alone. Plant-associated microbiota, in turn, can influence host water relations, nutrient acquisition, and hormonal status [76,77]. The relevance of this general framework to PRI is straightforward: by imposing wet–dry alternation across the root zone, PRI is likely to alter both the amount and composition of rhizodeposition, as well as the microscale chemical gradients that drive microbial recruitment. Thus, the importance of the rhizosphere under PRI is not limited to the barley study; it is also supported by a broader understanding of how root physiology and microbial assembly are coupled.

Several PRI-related studies support this interpretation indirectly through changes in rhizosphere function. In tomato, alternate PRI stimulated soil nitrogen mineralization and improved plant N nutrition under dry–wet cycles [68]. Subsequent work likewise reported that nutritional responses under PRD are shaped by drying–rewetting dynamics, indicating that the rhizosphere under PRI is functionally distinct from that under uniform irrigation [69]. In maize, APRI combined with biochar altered microbial respiration, microbial biomass N, soil N availability, and plant nitrogen use [67]. Even without full microbiome profiling, these studies show that PRI can alter rhizosphere function, particularly in nutrient release and acquisition. Root traits help mediate these effects. Greater root proliferation, higher root-to-shoot ratio, enhanced secondary root formation, and improved hydraulic conductivity all modify the physical interface through which exudates are released, and microbes interact with the plant [69,70,71,72]. Earlier work on PRI already recognized that rewetting stimulates new root growth and may renew the ability of roots to sense and respond to soil drying [25]. From a rhizosphere perspective, this renewed growth is doubly important: newly formed roots are typically more active in exudation and nutrient uptake, and therefore more influential in structuring the surrounding microbiome. Accordingly, rhizosphere responses under PRI are likely to arise through both bottom-up physicochemical effects of alternating moisture and root-mediated biological effects of altered growth and exudation [33]. At present, however, the relative contribution of these two routes cannot be separated with confidence. The most defensible interpretation is that root-metabolite reprogramming and microbiome shifts are coordinated responses within the same PRI-altered rhizosphere, rather than evidence of a demonstrated one-way causal chain from metabolites to microbial recruitment [47]. Direct causality would require experiments that decouple moisture heterogeneity from root exudation, such as sterile or gnotobiotic PRI systems, synthetic microbial communities, exudate tracing, microbiome-transfer assays, and targeted metabolite addition or depletion under matched wet–dry conditions. For this reason, the rhizosphere should not be treated simply as a passive background to PRI signaling [53]. However, it should also not yet be presented as a proven causal driver of PRI-induced acclimation. A more evidence-matched interpretation is that rhizosphere restructuring is an associated, potentially functional component of the PRI response, whose causal contribution remains to be directly tested. In particular, microbial shifts may be drivers, modifiers, or consequences of plant responses, and current datasets often cannot distinguish among these possibilities [67]. This limitation is especially important because many PRI microbiome studies remain based on controlled systems or single-crop examples, with limited evidence for reproducible field-scale microbial signatures. It is better understood as a dynamic component of the response system itself. Figure 4 reflects this logic by positioning root metabolism, root exudation, root-zone physicochemical conditions, and microbial assembly within a single conceptual interface rather than as disconnected topics.

### 6.3. From Association to Causality: What Is Still Unresolved

Despite this progress, the field remains at an early stage in moving from association to causality. This limitation is particularly important because microbiome-related results are often derived from correlation networks, taxonomic enrichment patterns, and co-occurrence analyses, which can identify candidate interactions but cannot establish causal direction [4,5,6,7]. Consequently, microbial shifts observed under PRI should not automatically be interpreted as drivers of plant acclimation; they may instead be consequences of altered root metabolism, exudation, root growth, moisture distribution, or soil physicochemical conditions [56]. The barley PRD study provides an unusually rich dataset, yet it also illustrates the main unresolved problem. PRD under low temperature was associated with increased glutathione and 9-octadecenamide in roots, altered glutathione-related gene expression, and the recruitment of bacterial and fungal modules, all of which were statistically linked to these metabolites [65]. These relationships are biologically plausible but constitute associative evidence. Network correlations, module–trait associations, and taxonomic enrichment can identify candidate interactions; they cannot establish whether metabolite changes drive microbial assembly, whether microbial shifts feedback on root metabolism, or whether both arise in parallel from the same PRD-imposed wet–dry environment [71]. The absence of significant chemotaxis of *Sphingobium quisquiliarum* or *Mortierella alpina* toward glutathione or 9-octadecenamide, together with the lack of significant enrichment of these taxa after metabolite treatment, argues against a simple model in which these metabolites directly recruit the candidate microbes [74]. A more rigorous interpretation is therefore that root-metabolite changes and microbiome shifts are associated components of a shared PRI response, most likely shaped by moisture heterogeneity, altered root physiology, exudation dynamics, oxygen availability, nutrient pulses, and microsite redox variation [38]. At present, the barley evidence supports coordination rather than causation: the root metabolome, transcriptome, and microbiome change in a biologically coherent manner under PRD, but the direction of control among these components remains unresolved [47]. Establishing causation will require perturbation-based experiments, including metabolite depletion or addition under sterile and non-sterile conditions, synthetic-community reconstruction, microbiome-transfer assays, and isotope tracing of exudate-derived carbon under matched wet–dry treatments [14,15,16,17]. Glutathione and 9-octadecenamide improved low-temperature tolerance when added externally, increasing Fv/Fm by 23.4% and 22.9% and reducing relative conductivity by 12.7% and 19.2%, respectively [65]. These metabolite-addition experiments support a functional role for glutathione and 9-octadecenamide in plant stress protection, but they do not demonstrate that these metabolites causally structure the microbiome. Similarly, microbial inoculation experiments may indicate that selected taxa can contribute to stress tolerance, but they do not prove that PRD-induced metabolites are responsible for recruiting those taxa in situ [34]. The evidence therefore supports functional relevance of selected metabolites and microbes, but not a resolved causal chain linking metabolite accumulation, microbial recruitment, and plant acclimation. However, neither *Sphingobium quisquiliarum* nor *Mortierella alpina* showed significant chemotaxis toward either metabolite, and their relative abundance in the rhizosphere did not increase significantly after metabolite treatment [65]. Conversely, inoculation with *S. quisquiliarum* and *M. alpina* increased Fv/Fm by 16.6% and 15.6%, respectively, and reduced relative conductivity by 18.6% and 17.14%; moreover, *M. alpina* significantly increased glutathione concentrations in roots and leaves by 29.1% and 14.7%, respectively [65]. These results indicate that both the metabolites and the microbes may contribute to the observed stress-acclimation phenotype. However, they do not establish whether metabolites act upstream of microbial assembly, whether microbial changes alter root metabolism, or whether both responses occur in parallel under the same wet–dry environment [70]. The mechanistic sequence is therefore more complex than “root metabolite attracts beneficial microbe, which then explains the phenotype.”

A second unresolved issue concerns experimental scale and treatment structure. Much of the strongest mechanistic evidence still comes from split-root pots, controlled environments, or PRI systems combined with co-treatments such as biochar or elevated CO_2_. These designs are informative, but they also complicate attribution. In tomato, PRI under elevated CO_2_ clearly altered root morphology and leaf stoichiometry [66], yet the study did not directly determine whether improved nutrient relations were mediated by altered exudation chemistry, rhizosphere turnover, or intrinsic developmental plasticity of roots. In maize, APRI combined with biochar clearly changed microbial activity, N availability, and root development [80], but the respective contributions of irrigation geometry and substrate modification cannot be fully separated. More work is therefore needed using designs that explicitly disentangle moisture heterogeneity, root metabolic state, exudation, and microbial function. A third unresolved issue is whether conserved belowground PRI mechanisms exist across crops and stresses. The barley system suggests a glutathione-centered, microbiome-linked mechanism under low temperature [65], but it remains unclear how widely that model generalizes. In other systems, PRI may operate more strongly through changes in root growth, nutrient turnover, or hydraulic conductivity than through the same metabolites or taxa [81]. For example, tomato and maize studies emphasize root morphological plasticity and nutrient acquisition [66,67], whereas the barley study emphasizes omics-based and rhizosphere-network responses [65]. The central question is therefore not whether PRI affects the root–rhizosphere interface—it clearly does—but which elements of that interface are conserved, which are stress-specific, and which are strongly dependent on crop type, soil, or treatment design.

Methodologically, this means the field now needs experiments that move beyond descriptive omics and network analysis. Time-resolved sampling across drying–rewetting cycles, isotope tracing of exudate-derived carbon and nutrient flow, gnotobiotic or synthetic-community systems, reciprocal microbiome transfer, and targeted perturbation of candidate metabolites will all be necessary if PRI research is to progress from ecological association to mechanism. This is one of the reasons why root-centered regulation represents such a strong source of novelty for the present review: it identifies a major part of the PRI response that is biologically important, conceptually rich, and still insufficiently resolved. Taken together, the available evidence supports a framework in which PRI modifies root metabolism, root architecture, physicochemical root-zone conditions, and rhizosphere assembly in parallel, with downstream consequences for nutrient cycling and plant stress resilience. Figure 4 summarizes this framework and makes clear that the causal links among its components remain a major frontier for future PRI research.

## 7. Translation to Agricultural Crops and Future Research Priorities

The central challenge for PRI research is no longer whether the treatment can alter plant physiology. That point is already established across fruit trees, vegetables, and field crops. The more important question is how PRI can be translated from mechanistic experiments into a predictive, reproducible, and agronomically useful framework for crop production under climatic uncertainty. If the field continues to report water-saving, partial stomatal closure, and improved water-use efficiency without defining where such responses are conserved, where they are conditional, and how they can be reproduced at the field scale, PRI will remain physiologically interesting but only partly translatable. This section, therefore, focuses on three linked issues: which PRI responses can be generalized across crops, which depend strongly on crop type, genotype, and irrigation design, and which research priorities should define the next generation of studies. These priorities are summarized in Table 4.

### 7.1. What Can Be Generalized Across Crops

Several PRI responses are now sufficiently consistent across species to be treated as general features rather than isolated observations. The first is the capacity of heterogeneous root-zone moisture to generate root-to-shoot signaling while maintaining water uptake from the wet compartment. This mechanistic principle has been reproduced across grapevine, potato, tomato, maize, cotton, and lemon systems [82,83,84,85,86]. Although the magnitude of the response varies, the core logic remains stable: drying roots generate signals associated with stomatal restraint and growth adjustment, whereas wet roots maintain hydraulic supply to the shoot. This basic structure is one of the few genuinely conserved PRI mechanisms and provides the foundation for any translational framework. A second generalizable feature is improved water-use efficiency, though not necessarily via identical physiological pathways in all crops. In grapevine, PRD improved water-use efficiency by up to 50% without a significant reduction in crop yield [28]. In potato, PRD reduced irrigation water use by about 30% and increased crop water-use efficiency by 58.7–59.0%, while maintaining tuber biomass similar to that under full irrigation [29]. In lemon, PRD increased crop water-use efficiency by 83%, even though the response could not be explained solely by leaf xylem ABA concentration [37]. Across crops, therefore, improved WUE is a robust agronomic output, but its mechanistic basis may vary among stronger stomatal control, altered leaf area development, improved hydraulic economy, and improved root acquisition.

A third broadly reproducible feature is the importance of alternation itself. PRI is not a static split between one wet and one dry compartment; it is a dynamic treatment in which switching frequency helps preserve the hydraulic and signaling competence of the drying side [26,34]. Without alternation, roots in the dry sector may progressively lose radial conductivity, reduce secondary-root activity, and weaken their contribution to xylem signaling [25]. This principle appears sufficiently stable across experimental systems to justify generalization. A fourth area of emerging generality is the link between PRI and improved coordination of plant responses under stress. In barley under low temperature, PRD improved net photosynthetic rate by 90.6%, stomatal conductance by 130%, and reduced relative conductivity by 45.2% relative to full irrigation under the same stress [52]. In cotton and maize, PRI-related treatments improved water relations, nutrient balance, and root function under salinity or drought stress [58,59]. The most defensible generalization is therefore not that PRI activates exactly the same downstream pathway in all species, but that it improves the coupling among water status, signaling, metabolism, and resource acquisition under constraint [87]. This distinction is important for a mature review. Conserved outcomes and conserved mechanisms are not equivalent. ‘PRI often improves WUE’ is a robust statement. ‘PRI operates through the same downstream signaling network in all crops’ is not. A publication-grade synthesis must therefore separate repeatable agronomic outputs from deeper mechanistic claims.

The most defensible generalization is therefore not that PRI activates exactly the same downstream pathway in all species, but that it improves the coupling among water status, signaling, metabolism, and resource acquisition under constraint [87]. This distinction is important for a mature review. Conserved outcomes and conserved mechanisms are not equivalent. ‘PRI often improves WUE’ is a robust statement. ‘PRI operates through the same downstream signaling network in all crops’ is not. A publication-grade synthesis must therefore separate repeatable agronomic outputs from deeper mechanistic claims. Cross-crop evidence is strongest when studies use shared physiological readouts, including stomatal conductance, transpiration, net photosynthesis, leaf water potential, relative water content, sap-flow partitioning, WUE, biomass allocation, yield, and quality traits [24,25,26,27,28]. Across grapevine, potato, tomato, maize, cotton, barley, and citrus, recurrent outcomes include improved WUE, moderated stomatal conductance, partial maintenance of photosynthesis, improved plant water status, and reduced stress injury [42]. However, the mechanistic interpretation differs among crops. In grapevine, citrus, sunflower, and tomato, the emphasis has mainly been on ABA transport, sap flow delivery, hydraulic status, cytokinin balance, and stomatal regulation [48]. In barley, recent evidence extends the comparison to antioxidant activity, glutathione metabolism, root transcriptomic responses, and rhizosphere restructuring under low temperature. In maize and cotton, PRI-related responses are more often interpreted in terms of root function, nutrient uptake, ion balance, hydraulic conductance, antioxidant enzymes, and interactions with soil amendments [12,13,14,15,16]. Thus, the current literature supports partial conservation at the physiological level, but not yet universal conservation at the molecular or rhizosphere level.

### 7.2. What Depends on Crop Type, Genotype, and Irrigation Design

Many PRI responses remain strongly contingent on crop identity, genotype, canopy structure, soil properties, and irrigation design. Crop type matters because the translation of root-sourced signaling into whole-plant water use differs among canopies [88]. Kang and Zhang emphasized that stomatal control over transpiration may differ between dense field canopies, such as wheat and maize, and more sparsely arranged fruit trees, because canopy boundary resistance can buffer or dilute the impact of stomatal closure on whole-plant water loss [25]. The same root-sourced signal may therefore produce different water-use outcomes depending on crop architecture. In dense canopies, stomatal regulation may be only one component of transpiration control; in fruit trees, the same signal may have more direct effects on canopy water loss. Genotype introduces a second level of contingency [89]. PRI responses depend on inherent variation in root distribution, stomatal sensitivity, hydraulic conductance, hormonal balance, and sink regulation. Tomato studies under elevated atmospheric CO_2_ have already shown genotype-dependent responses of physiology and fruit quality under PRI [54]. These crop- and genotype-dependent responses highlight why future comparative studies should use a common molecular and biochemical readout set rather than relying solely on crop-specific endpoints [10]. At minimum, cross-crop PRI studies should pair physiological measurements with ABA and cytokinin dynamics, sap-flow-weighted ABA export, root hydraulic conductance, aquaporin expression, antioxidant enzymes, ROS or lipid-peroxidation markers, osmolytes such as proline and soluble sugars, nutrient-transporter markers, and selected root transcripts linked to ABA signaling, redox regulation, osmotic adjustment, and nutrient acquisition [65]. Where rhizosphere mechanisms are proposed, these should be complemented by standardized metabolomic and microbiome profiling. Without such harmonized readouts, similar outcomes across crops may be mistaken for conserved mechanisms, even when they arise through different hydraulic, hormonal, metabolic, or root architectural routes [80]. Potato studies likewise emphasized that ABA-mediated stomatal control and leaf area restraint can support improved WUE but also identified unresolved questions regarding the causal link between xylem ABA concentration and stomatal conductance under PRD [90]. In crops with contrasting inherent ABA sensitivity, the same PRI design may therefore produce different balances among stomatal regulation, growth restraint, and yield maintenance.

Irrigation design introduces another major source of variation. Switching frequency, wet:dry root-zone ratio, irrigation delivery system, rooting volume, and soil type all influence PRI signaling efficiency [25,26]. Dodd and colleagues showed that the fraction of sap flow contributed by drying roots declines with increasing dryness, and that once it falls below roughly 20% of total sap flow, ABA export to the shoot may decline despite high local ABA concentration [26]. This has direct translational consequences: switching too slowly can reduce signal export, whereas switching too rapidly may prevent the drying side from generating a sufficiently strong signal. The physiologically appropriate threshold for alternation still remains insufficiently defined. Field-scale reproducibility also remains uneven. While PRI has shown strong results in controlled systems and in several field studies, not all crops respond equivalently under commercial conditions. Potato and grapevine provide some of the strongest positive field cases [28,29], while work in cotton, olive, bean, and tomato indicates that outcomes vary with stress intensity, climate, and the specific comparison used, especially PRI versus regulated deficit irrigation (RDI) or conventional deficit irrigation [85,86,87,88]. In common bean, for example, PRD and RDI both improved water-use efficiency relative to full irrigation, but the difference between them was not always large [87]. This is a crucial translational point: PRI must be evaluated not only against full irrigation, but against water-matched DI or RDI controls; the specific biological value of root-zone heterogeneity cannot be isolated. One of the most significant design-dependent issues is whether PRI is genuinely distinct from generic mild drought. If PRI is compared only with full irrigation, then any observed advantage may reflect reduced water supply or mild stress exposure rather than a specific effect of root-zone heterogeneity. The strongest PRI studies are therefore those that compare full irrigation, DI/RDI, and heterogeneous root-zone drying simultaneously, allowing one to distinguish between the amount of water and its distribution pattern.

### 7.3. Research Priorities for Next-Generation PRI Studies

The next phase of PRI research should be defined less by the accumulation of additional crop-specific case studies and more by the resolution of specific mechanistic and translational uncertainties. These can be organized into several priority themes, summarized in Table 4. The first priority is to distinguish PRI from a generic mild drought more rigorously. Many of the most frequently cited benefits of PRI—improved WUE, partial stomatal closure, and modest yield maintenance—can also arise under carefully managed DI. The critical unresolved issue is therefore not whether PRI outperforms full irrigation, but whether spatial heterogeneity per se adds mechanistic and agronomic value beyond what can be achieved with whole-root-zone deficit irrigation. This requires experiments that match total water input while varying only the spatial arrangement of wet and dry zones. The second priority is to resolve the relative roles of ABA-dependent and ABA-independent signaling. ABA is clearly central to PRI, yet several studies already show that leaf-level responses cannot always be explained by leaf xylem ABA concentration alone [26,37]. The field, therefore, needs integrative experiments that combine xylem sap profiling, sap-flow partitioning, hydraulic measurements, and shoot sensitivity assays across genotypes with contrasting ABA responsiveness. A key goal should be to determine when PRI responses are dominated by hormone flux, when they depend more strongly on hydraulic modulation, and when both are embedded in broader signaling networks involving cytokinins, calcium, reactive oxygen species, jasmonates, or redox metabolites [43,44].

The third priority is field-scale reproducibility. Mechanistically elegant studies often depend on split-root pots, controlled greenhouses, rainout shelters, or highly managed drip systems. These remain indispensable for discovery, but the field also needs designs that retain mechanistic resolution under field conditions [55]. That means more studies linking soil moisture mapping, root-zone heterogeneity, sap-flow partitioning, canopy gas exchange, yield formation, and quality outcomes in real cropping systems. Reproducibility at the field scale is not simply a final validation step; it is part of the mechanistic question itself. The fourth priority is to separate crop-specific from conserved mechanisms through coordinated cross-crop comparison. At present, the field has enough data to propose that root-sourced signaling, improved WUE, and stress coordination are broadly conserved outcomes of PRI, but not enough to define which downstream pathways are universal [22]. Future studies should therefore compare multiple crops under matched PRI, DI, and full-irrigation treatments using shared physiological and molecular readouts. These comparisons should also include hormone-centered markers beyond ABA alone. In particular, auxin-related readouts, including IAA levels, auxin transport markers such as PIN and AUX/LAX genes, Aux/IAA and ARF expression, GH3-mediated auxin conjugation, and auxin–ABA or auxin–ROS interaction markers, would help determine whether PRI modifies root growth and stress acclimation through broader hormonal crosstalk [48,49,50,51]. Incorporating such markers would align PRI research with recent auxin biodynamics frameworks and would help distinguish conserved physiological outcomes from crop-specific hormonal mechanisms. The physiological core should include soil moisture distribution, sap-flow partitioning, stomatal conductance, transpiration, net photosynthesis, leaf water potential, relative water content, root hydraulic conductance, biomass allocation, WUE, yield, and quality [6,7,8,9]. The molecular and biochemical core should include xylem/root ABA, cytokinin status, aquaporin expression, antioxidant enzymes, glutathione-related markers, ROS and MDA, proline, soluble sugars, nutrient-transporter expression, and selected root transcripts associated with ABA, redox, osmotic, and nutrient-acquisition pathways. This shared framework would allow the field to determine whether PRI produces conserved mechanisms across crops or only similar agronomic outcomes through crop-specific physiological routes. Glutathione-centered root reprogramming may be especially relevant in cold-stressed barley [65], whereas root architectural plasticity and nutrient acquisition may be more central in tomato or maize [66,67]. Cross-crop comparative studies using shared physiological and omics readouts are now needed to distinguish common PRI modules from crop- or stress-specific variants. A related priority is to move beyond bulk tissue omics toward high-resolution stress-biology approaches. Recent single-cell/nucleus RNA sequencing and spatial transcriptomics frameworks show that plant stress responses are spatially and cell-type specific, and that bulk RNA analyses can mask important heterogeneity among tissues and cell populations [75]. Applying these approaches to PRI would allow future studies to identify which root cell types sense localized drying, which vascular or xylem-associated cells regulate long-distance signaling, which cell populations undergo antioxidant or osmotic reprogramming, and which rhizosphere-facing tissues participate in exudation and microbial interactions [75]. Such a resolution would strengthen mechanistic interpretation and help explain why conserved physiological outcomes do not always correspond to conserved molecular mechanisms across crops [80]. The fifth priority is microbiome causality and field reproducibility. Section 6 indicates that rhizosphere restructuring is an emerging dimension of PRI research, but current evidence remains largely associative, especially in the barley root–microbiome example. In addition, the reproducibility of PRI-associated microbial shifts under field conditions remains poorly characterized [34]. Most available evidence is still derived from controlled environments, specific crop systems, or individual stress contexts, making it difficult to determine whether observed microbial signatures are generalizable across soils, climates, irrigation designs, and crop species. Future work must separate three levels of evidence: correlation between metabolites and microbial modules, functional effects of metabolites or microbes on plant stress tolerance, and direct causation showing that one component drives the other in situ. The manuscript therefore treats the microbiome’s involvement as a promising but unresolved component of PRI biology, rather than an established causal pathway. Synthetic community experiments, microbiome transfer, isotope tracing of exudate-derived substrates, targeted manipulation of candidate metabolites, and replicated field microbiome surveys should now become part of the PRI toolbox. Field-scale studies should combine microbial profiling with soil moisture mapping, root-zone physicochemical measurements, exudate or metabolite analysis, crop performance, and matched PRI–DI controls. Without these approaches, microbiome responses will remain descriptive rather than explanatory, and their reproducibility under agronomic conditions will remain uncertain [83,91].

The sixth priority is standardization of switching protocols. Farmers and researchers still lack clear, physiology-based guidance on when alternation should occur. Existing evidence suggests that switching too late reduces signal export because drying roots contribute insufficient sap flow, whereas switching too early may weaken signal intensity [27]. Priority experiments should therefore define alternation thresholds using measurable criteria, such as the dry-side fraction of transpirable soil water, sap-flow contribution, root water potential, or xylem ABA flux, rather than calendar time alone [92]. The seventh priority is the explicit study of multi-stress environments. PRI is increasingly discussed as a priming-like treatment capable of engaging overlapping stress pathways. However, most studies still examine a single dominant stress at a time and do not always distinguish acclimation during PRI from true priming for a later stress event. Future work should therefore include designs with a defined priming phase, a recovery or memory interval, and a subsequent stress challenge, while retaining full-irrigation and matched deficit-irrigation controls [5,6,7,8]. More factorial studies are needed that combine drought with salinity, low temperature, heat, or elevated CO_2_, while maintaining an explicit comparison between PRI and DI. Heat stress deserves separate experimental attention because current PRI evidence rarely isolates temperature as an independent factor [62]. Future heat-focused studies should manipulate air or canopy temperature independently of soil water supply and vapor-pressure deficit, and should compare full irrigation, matched DI, and PRI under both control and elevated-temperature conditions. Shared thermal-response readouts should include canopy temperature, leaf energy balance, stomatal conductance, transpiration, Fv/Fm, electrolyte leakage, MDA, antioxidant enzymes, aquaporin expression, heat-shock protein expression, pollen viability, fruit set, and yield stability. Such designs would help determine whether PRI confers true heat-stress tolerance or mainly improves performance under heat-associated evaporative demand [73]. This is particularly important because future agriculture will increasingly be shaped by concurrent rather than isolated stresses [63,64]. The eighth priority is biomarker development. If PRI is to become predictable and scalable, the field will need measurable indicators that report whether the treatment is operating effectively. Candidate biomarkers include xylem ABA flux, sap-flow contribution of drying roots, dry-side soil water thresholds, stomatal sensitivity indices, glutathione status, specific metabolite markers such as 9-octadecenamide, and selected rhizosphere signatures [26,65]. Biomarker development would help move PRI from descriptive physiology toward diagnostic irrigation management. For these reasons, the future of PRI research lies not in simply adding more crop examples, but in designing experiments that identify decision rules, mechanistic boundaries, and predictive indicators. PRI will become a mature framework only when it can explain not just that plants respond, but which crops respond, under which designs, through which mechanisms, and with which measurable indicators of success.

**Table 4:** Research gaps and next-generation priorities in partial root-zone irrigation research.

Unresolved Question	Why It Matters	Present Limitation	Priority Experiment or Analytical Approach	References
PRI vs. generic mild drought	Essential for establishing whether root-zone heterogeneity adds value beyond reduced water supply.	Many studies compare PRI only with full irrigation rather than water-matched DI or RDI.	Use full factorial FI–DI/RDI–PRI designs at matched water input; include yield, WUE, sap-flow partitioning, and shoot/root signaling outputs.	[25,29,86,87]
ABA-dependent vs. ABA-independent signaling	Determines whether PRI is transferable across crops with contrasting hormone sensitivity.	Leaf responses are not always explained by leaf xylem ABA concentration alone.	Combine xylem sap profiling, sap-flow partitioning, hydraulic traits, and ABA-sensitivity genotypes; include cytokinin and redox measurements.	[26,27,37,42,43]
Field-scale reproducibility	Required for translation from mechanism to practice.	Strong mechanistic evidence often comes from split-root pots or greenhouse systems.	Couple field PRI trials with soil-moisture mapping, sap flow, canopy physiology, quality traits, and economic analysis across seasons and sites.	[28,29,86,88]
Crop-specific vs. conserved mechanisms	Needed to distinguish general PRI biology from crop-specific responses.	Cross-crop studies rarely use shared mechanistic measurements.	Run comparative experiments across fruit and agronomic crops using common physiological, biochemical, and omics markers.	[25,52,65,66,67]
Microbiome causality	Could redefine PRI from a shoot-centered to a root–rhizosphere-centered framework.	Current evidence is mostly correlative or network-based.	Use synthetic communities, microbiome transfer, gnotobiotic systems, exudate tracing, and targeted metabolite perturbation.	[65,73,75,77,81]
Standardized switching protocols	Essential for practical use and comparability among studies.	Switching is often based on arbitrary time intervals rather than physiological thresholds.	Define alternation thresholds using dry-side fraction of transpirable soil water, sap-flow contribution, root water potential, or xylem ABA flux.	[25,26,34]
Multi-stress experiments	Future cropping systems will face drought plus salinity, cold, heat, and elevated CO_2_ simultaneously.	Most PRI studies still focus on single dominant stresses.	While maintaining water uptake from the wetted side with salinity, low temperature, heat, and elevated CO_2_ while retaining DI comparators and shared endpoints.	[52,54,55,63,64]
Biomarker development	Needed to make PRI predictable, diagnosable, and scalable.	No agreed set of physiological or biochemical markers currently defines effective PRI.	Evaluate xylem ABA flux, dry-side sap-flow contribution, glutathione status, metabolite markers, and rhizosphere signatures as predictive indicators.	[26,65]

### 7.4. Challenges in Translating PRI to Commercial Field Conditions

Although PRI has strong mechanistic and agronomic potential, its translation from controlled experiments to commercial field conditions remains challenging. In split-root, pot, and greenhouse studies, wet and dry root compartments can be created with relatively high precision. In commercial fields, however, this spatial control is much more difficult to maintain. Soil heterogeneity is one of the main barriers. Variation in soil texture, structure, compaction, infiltration rate, drainage, salinity distribution, bulk density, and organic matter content can strongly influence how water moves through the root zone. In coarse-textured soils, the drying compartment may lose hydraulic connectivity too rapidly, whereas in heavy or poorly drained soils, lateral water movement may weaken the wet–dry contrast required for PRI signaling. Therefore, commercial PRI cannot be treated simply as reduced irrigation; it requires verification that a stable, yet non-lethal, wet–dry contrast is actually being created around active roots. Irrigation-system design is equally important. Successful PRI depends on controlled spatial placement of water, not only on reducing total irrigation volume. Drip-line position, emitter spacing, alternate-furrow layout, flow rate, irrigation duration, and distribution uniformity all determine whether one part of the root system remains hydrated while another part dries sufficiently to generate root-derived signals. If water spreads too widely, the dry compartment may not develop; if water placement is too restricted, the dry-side roots may become physiologically inactive. Field studies should therefore report irrigation layout, wetting pattern, soil moisture distribution at multiple depths, and the uniformity of water application, rather than describing PRI only in terms of irrigation percentage or switching interval.

Switching frequency is another critical implementation variable. PRI requires the dry side to remain dry long enough to generate a signal, but not so long that roots lose hydraulic connectivity and signal export capacity. Overly frequent switching may reduce the signaling contrast between root compartments, whereas delayed switching may convert PRI into damaging localized drought. The optimal switching interval is therefore unlikely to be universal. It should depend on soil type, crop species, rooting depth, evaporative demand, phenological stage, and irrigation method. Future commercial-scale studies should test threshold-based scheduling using soil moisture, root-zone water status, canopy temperature, leaf water potential, or sap-flow indicators, rather than relying only on fixed calendar-based alternation. Rooting depth and crop architecture further determine whether PRI can be implemented reliably. Shallow-rooted crops may respond rapidly to wet–dry alternation, but they may also be more vulnerable to excessive dry-side dehydration. Deep-rooted perennial crops may maintain water uptake more effectively, but their large and spatially complex root systems make it more difficult to impose a controlled wet–dry contrast. Root distribution also changes with soil depth, planting density, developmental stage, and long-term irrigation history. Therefore, commercial PRI studies should assess active root distribution, root length density, root activity, sap-flow partitioning between wet and dry zones, soil moisture at different depths, canopy water status, yield components, and quality traits. Without these measurements, it is difficult to know whether the crop is truly experiencing PRI or only a general reduction in water supply.

Economic feasibility is also essential for adoption. PRI may require additional irrigation infrastructure, separate control of wet and dry zones, soil-water sensors, automation, labour for switching, and decision-support tools. These costs must be balanced against measurable benefits such as irrigation water savings, yield stability, improved fruit or grain quality, nutrient-use efficiency, and resilience under stress. PRI may be more immediately attractive in high-value horticultural crops, where improved quality and water savings can justify additional management costs. In broad-acre field crops, adoption will require simpler delivery systems, robust scheduling rules, low-cost monitoring, and clear evidence that PRI provides advantages over matched deficit irrigation under commercial management. Thus, future field studies should include not only physiological and yield measurements but also infrastructure requirements, labor demand, management complexity, cost–benefit analysis, and seasonal risk assessment.

## 8. Conclusions

PRI should be regarded as more than a reduced-water irrigation practice only when its effects are evaluated against appropriately matched uniform deficit-irrigation controls. The strongest mechanistic evidence supports an integrated hydraulic–chemical signaling model in which dry-side ABA production, sap-flow-dependent ABA delivery, root water potential, and hydraulic connectivity jointly regulate stomatal behavior and downstream acclimation. Recent hormone-centered stress-regulation literature further suggests that PRI should be interpreted within a broader signaling network in which auxin biodynamics may influence root architecture, growth–defense balance, and stress resilience alongside ABA, cytokinins, ROS, and jasmonate signaling. The evidence synthesized in this review supports the view that PRI functions as a spatially structured treatment in which localized wet–dry partitioning can generate root-derived signals while maintaining water uptake from the wetted side. Through this organization, PRI can regulate transpiration, sustain photosynthetic performance, maintain membrane stability, and strengthen biochemical defense without imposing the same degree of generalized dehydration as whole-root-zone deficit irrigation. However, many responses associated with PRI, including ABA accumulation, osmotic adjustment, antioxidant activation, and stomatal regulation, also occur under conventional deficit irrigation. Therefore, the distinctiveness of PRI lies less in the identity of these response modules than in their spatial coordination, hydraulic buffering, signal-delivery context, and performance relative to matched DI.

Current evidence is strongest for drought, whereas responses to low temperature, salinity, heat-associated evaporative demand, and combined stresses remain more context-dependent. Recurrent improvements in WUE, stomatal regulation, photosynthetic maintenance, and yield stability should be interpreted as physiological convergence unless shared molecular, hormonal, hydraulic, and biochemical readouts demonstrate that the same mechanisms operate across crops. Future studies should therefore quantify the incremental contribution of wet–dry spatial partitioning above the baseline effect of mild deficit irrigation. Root-centered regulation is among the most promising directions for advancing PRI research. Recent studies suggest that PRI can modify root metabolism, transcript abundance, root architecture, nutrient acquisition, and rhizosphere organization. However, the causal architecture of this belowground response remains unresolved. Current evidence supports coordinated metabolite and microbiome responses under PRI but does not prove that root-metabolite shifts directly cause microbiome restructuring or that microbial shifts drive plant acclimation. These responses may instead arise partly in parallel from PRI-induced changes in root physiology, exudation, oxygen availability, nutrient turnover, redox conditions, and wet–dry soil microsites. Field-scale reproducibility of PRI-associated microbiome patterns also remains insufficiently characterized.

Future progress will depend on resolving PRI-specific effects from generic mild drought, clarifying ABA-dependent and ABA-independent pathways, standardizing switching thresholds, strengthening field-scale reproducibility, testing multi-stress environments, and developing predictive biomarkers of effective PRI performance. High-resolution approaches, including single-cell/nucleus RNA sequencing and spatial transcriptomics, will be important for determining whether PRI-associated root signaling, metabolic reprogramming, and rhizosphere interactions are controlled by specific root tissues or cell types rather than by uniform whole-root responses. Commercial translation will require field-scale evidence that a reliable wet–dry root-zone contrast can be maintained across heterogeneous soils, rooting depths, irrigation system designs, switching schedules, and economically realistic management conditions. In this context, PRI emerges as a promising, mechanistically informed strategy for climate-resilient crop management rather than simply an alternative irrigation schedule. Nevertheless, several proposed mechanisms, especially heat-stress responses, microbiome-mediated effects, and true stress-priming memory, remain provisional and require direct experimental validation.

## Data Availability

The original contributions presented in this study are included in the article. Further inquiries can be directed to the corresponding authors.
